# Novel edaravone-based azo dyes: efficient synthesis, characterization, antibacterial activity, DFT calculations and comprehensive investigation of the solvent effect on the absorption spectra†

**DOI:** 10.1039/d0ra06934e

**Published:** 2020-09-29

**Authors:** Mohammad Amin Davasaz Rabbani, Behzad Khalili, Hamid Saeidian

**Affiliations:** Department of Science, Payame Noor University (PNU) P. O. Box: 19395-4697 Tehran Iran; Department of Chemistry, Faculty of Sciences, University of Guilan P. O. Box 41335-1914 Rasht Iran b.khalili@guilan.ac.ir

## Abstract

The present study deals with designing and synthesizing novel dyes using the drug combination of edaravone and azo compounds which can be used as an indicator for anions and cations. The desired product synthesis was accomplished *via* a two-step process involving diazotizing the aromatic amines followed by the resultant salts coupling with edaravone. The resulting dyes were obtained with high yields under mild conditions. The structures of the dyes were identified with UV-vis, FT-IR, ^1^H NMR and ^13^C NMR spectra and CHN analysis. To investigate the solvatochromism effect, the interaction of different solvents with the selected dyes was evaluated using several parameters including the dielectric constant, refractive index, hydrogen bond donating ability, hydrogen bond accepting ability and dipolarity/polarizability scale. To achieve deep understanding about the stability and geometrical characteristics of the azo–hydrazo tautomers of the synthesized dyes and their UV-visible spectra prediction, some DFT calculations were also carried out on the synthesized dyes. The antibacterial activities of some synthesized compounds were also evaluated using the disk diffusion method. The results revealed different activity of the selected synthesized dyes for antibacterial tests against selected Gram positive and Gram negative bacteria.

## Abstract 2

The present study deals with designing and synthesizing novel dyes using the drug combination of edaravone and azo compounds which can be used as an indicator for anions and cations.

## Graphical abstract

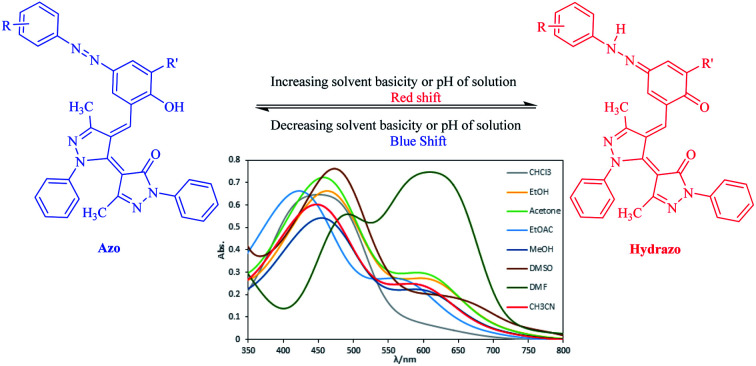

## 1. Introduction

The world around us contains a variety of colors. Humans have been using color in their lives for thousands of years. The earliest example of color usage is related to pre-historic paintings on cave walls. Until the mid-19th century, most colors were extracted from plants and animals through a laborious process. In 1845, a young chemist named Perkin decided to chemically synthesize a natural substance called quinine, which was thitherto used to treat malaria. He arrived at a slim purple substance through combining aniline with potassium dichromate which leads to an attractive purple solution after adding alcohol.^1^ Thus, the first artificial color called Mauveine was synthesized.^2^ Azo dyes comprise the largest family of the organic colors, accounting for more than 50% of the commercial dyes.^3^ These important discoveries led to the development of azo dyes, which are now being considered as the most important group of the organic dyes.^4–8^ These dyes contain one azo group (–N=N–) in their structures but can be in the form of diazo, triazo, tetraazo or more (polyazo). In addition to using these compounds as color, the azo dyes are implemented in many industrial processes such as printing, textile, photography, color additives, medicine and molecular diagnosis.^9–13^ The azo group is typically linked to the benzene rings, naphthalene and in some cases aromatic heterocycles such as pyrrole. The azo dyes are highly colorant and one can synthesize dyes with yellow, orange, red, blue or even green colors depending on the selection of the molecule's structure. The multifunctional use of azo compounds can be attributed to several advantages such as easy and inexpensive preparation, high consistency colors creation, inexpensive and affordable raw materials.

In recent years, a number of investigations have been carried out on the design and synthesis of azo compounds as the colorimetric sensors.^14–19^ Khanmohammadi *et al.* have conducted a research on the design and synthesis of new naphthalene-based azo–azomethine dyes. The sensory feature of the synthesized compounds for rapid detection of inorganic fluoride over the other anions, such as Cl^−^, Br^−^, I^−^ and AcO^−^, in DMSO/water media was tested. The results showed that the dyes can detect F^−^ even at 2.04 × 10^−6^ M level.^20^ In another study, Mohammadi *et al.* have prepared eight thiazolidinone derivatives as biologically active dyes with antibacterial properties. Positive solvatochromism was observed with an increment in the solvents polarity for the synthesized dyes.^10^ In 2016, a new quinazolinone-based dye was designed and synthesized for rapid and selective Cu^2+^ detection.^21^

Synthesis of the nitrogen-containing heterocyclic scaffolds has always been an important area of interest in chemical research, as these groups meet wide range of applications in the materials science, catalysts, color chemistry and most importantly, drug chemistry. Among heterocyclic compounds containing nitrogen atoms, pyrazole and its derivatives are used as important building blocks for the pharmaceutical compounds and possess a wide variety of biological activities.^22^ Edaravone (5-methyl-2-phenyl-2,4-dihydro-3*H* pyrazol-3-one) is a drug with the formula C_10_H_10_N_2_O and brand name of Radicut being used as the treatment for amyotrophic lateral sclerosis (ALS) which is a kind of progressive neurological disease.^23^ The performance mechanism of edaravon is not completely identified. It is also known as an antioxidant.^24^ Dyes based on 5-pyrazolone, are especially extensively used in color photographic materials.^25–27^

In the context of our general interest in the synthesis of heterocyclic compounds,^28–41^ herein, we propose a facile synthesis of the new dyes based on edaravone due to the unique properties of this compound. The synthesis of the desired compounds is possible through the coupling reaction of azo compounds with edaravone (D1–D10). In the following, the absorption spectra of four selected compounds of dyes (D1–D4) were measured in eight solvents having different polarities. The solvent dependent maximum absorption band shifts were evaluated using the Kamlet–Taft polarity parameters including hydrogen bond donating ability (*α*), hydrogen bond accepting ability (*β*) and dipolarity/polarizability polarity scale (π*), together with the dielectric constant (*ε*) and refractive index (*n*). It should be noted that the simplest solvent effect on the dye is the solvatochromism one observed by Hantzschlater referring to the influence of solvent on the position, intensity and shape of the spectral bands of the different compounds.^42,43^ When the shifts towards the higher wavelengths with increasing solvent polarity, the bathochromism phenomenon occurs. Whereas hypsochromism refers to the dye's maximum wavelength shifting towards the lower wavelengths with an increment in the solvent polarity. Solvatochromism is a powerful tool for investigating the physical and chemical properties of the molecules in the liquid phase, yet one of the most challenging researches due to its complexities for the chemists.

## 2. Experimental

### 2.1 General information

All chemicals used in this study were prepared from Sigma-Aldrich (St. Louis, MO, USA), Fluka (Neu-Ulm, Germany) and Merck (Darmstadt, Germany) companies. The progress of the reactions was tracked by thin-layer chromatography (TLC) using aluminum plates coated by silica gel 60 F254. The FT-IR spectra were provided using Shimadzu FT-IR4100 setup through preparing the samples by KBr tablet. The UV-vis for the synthesized dyes were obtained *via* the Rayleigh UV-1800 setup. The melting points of the dyes (D1–D10) were measured using IA9000 electric melting apparatus. A Bruker (DRX-500 Avanes) NMR was used to record the ^1^H NMR and ^13^C NMR spectra (ESI†). All NMR spectra were determined in DMSO at room temperature. The molecular weight of some products D1–D10 were determined by using LC-MS technique. LC-MS analysis was performed on a Shimadzu LCMS 2010 A by using positive electrospray ionization.

### 2.2 General procedure for synthesis of the azo compounds 4

Aniline derivatives 1 (1 mmol) were poured in a 100 mL flask equipped with a magnetic stirrer and then placed into the water-ice bath to reach a temperature of 0–4 °C. Then, a mixture of 3 mmol of concentrated sulfuric acid was prepared in 10 mL of cold distilled water and added to the amine solution within 5–10 minutes. After that, 1 mmol of sodium nitrite dissolved in 3 mL of distilled water was added while the temperature was maintained below 4 °C within 20–30 minutes. The prepared diazonium salt solution was immediately used in the next step to synthesize the corresponding azo compounds 4. To a stirred suspension of 2-hydroxybenzaldehyde 3 (1 mmol), potassium carbonate (5 mmol) and sodium hydroxide (2 mmol) in water, the prepared diazonium salt 2 was added dropwise at 0–4 °C. After 2 hours, pH was adjusted to 5.5 by adding hydrochloric acid. The resulted precipitate was filtered off, washed and dried as well.

### 2.3 General procedure for the synthesis of the desired dyes D1–D10

In a 10 mL flask equipped with the magnetic stirrer, edaravone (2 mmol) and the synthesized azo compounds 4 (1 mmol) are dissolved in acetonitrile (5 mL), DABCO (2 mmol) is added to the solution and stirred at 0–4 °C for 3 hours. After the reaction completion, the resulted precipitate was filtered off and recrystallized in ethanol for further purification.

### 2.4 Spectral data for the desired dyes D1–D10

Product D1: orange solid, yield: 77%, mp: 202–204 °C; FT-IR (KBr, *ν*/cm^−1^): 3411, 2958, 2930, 2876, 1729, 1641, 1597, 1496, 1283, 774, 753.^1^H NMR (400 MHz, DMSO-*d*_6_) *δ*: 8.16 (d, *J* = 2.2 Hz, 1H), 7.95 (dd, *J* = 8.8, 1.2 Hz, 4H), 7.72 (d, *J* = 8.4 Hz, 2H), 7.35–7.29 (m, 6H), 7.22 (d, *J* = 2.2 Hz, 1H), 7.04 (t, *J* = 7.4 Hz, 2H), 5.14 (s, 1H), 3.86 (s, 3H), 2.39 (s, 3H), 2.18 (s, 6H). ^13^C NMR (100 MHz, DMSO-*d*_6_) *δ*: 158.2, 150.6, 148.2, 146.7, 141.4, 140.5, 134.1, 130.3, 129.0, 128.7, 124.9, 123.2, 122.5, 119.5, 118.4, 102.1, 97.2, 56.2, 28.4, 21.4, 13.7. Anal. calcd for C_35_H_30_N_6_O_3_: C 72.15, H 5.19, N 14.42, found: C 72.01, H 5.34, N 14.23. MS (ESI): 583 [M + H]^+^. Product D2: brown solid, yield: 87%, mp: 176–178 °C; FT-IR (KBr, *ν*/cm^−1^): 3413, 2959, 2930, 1732, 1640, 1617, 1519, 1497, 1344, 1283, 1238, 772. ^1^H NMR (400 MHz, DMSO-*d*_6_) *δ*: 8.33 (d, *J* = 8.8 Hz, 2H), 8.08 (d, *J* = 1.8 Hz, 1H), 7.97 (d, *J* = 7.6 Hz, 4H), 7.85 (d, *J* = 8.8 Hz, 2H), 7.33 (t, *J* = 8.0 Hz, 4H), 7.25 (d, *J* = 1.8 Hz, 1H), 7.06 (t, *J* = 7.4 Hz, 2H), 5.09 (s, 1H), 3.85 (s, 3H), 2.20 (s, 6H). ^13^C NMR (100 MHz, DMSO-*d*_6_) *δ*: 158.2, 154.7, 150.4, 146.7, 145.8, 143.3, 141.3, 136.2, 129.4, 128.8, 125.7, 123.3, 120.9, 119.5, 118.5, 101.7, 97.0, 56.1, 28.3, 13.6. Anal. calcd for C_34_H_27_N_7_O_5_: C 66.55, H 4.44, N 15.98, found: C 66.73, H 4.30, N 15.72. MS (ESI): 614 [M + H]^+^. Product D3: yellow solid, yield: 80%, mp: 176–177 °C; FT-IR (KBr, *ν*/cm^−1^): 3412, 2959, 2930, 2874, 1729, 1640, 1598, 1499, 1278, 775. ^1^H NMR (400 MHz, DMSO-*d*_6_) *δ*: 8.40 (d, *J* = 2.4 Hz, 1H), 7.95 (d, *J* = 8.0 Hz, 4H), 7.71 (d, *J* = 8.4 Hz, 2H), 7.50 (dd, *J* = 8.6, 2.4 Hz, 1H), 7.37 (d, *J* = 8.4 Hz, 2H), 7.31 (t, *J* = 8.0 Hz, 4H), 7.04 (t, *J* = 7.4 Hz, 2H), 6.85 (d, *J* = 8.6 Hz, 1H), 5.07 (s, 1H), 2.69 (q, *J* = 7.4 Hz, 2H), 2.18 (s, 6H), 1.25 (t, *J* = 7.4 Hz, 3H). ^13^C NMR (100 MHz, DMSO-*d*_6_) *δ*: 158.3, 157.6, 150.9, 146.6, 145.5, 141.4, 134.1, 129.1, 128.9, 128.7, 123.2, 122.6, 122.0, 119.5, 118.5, 115.6, 102.1, 28.5, 15.8, 13.6. Anal. calcd for C_35_H_30_N_6_O: C 74.19, H 5.34, N 14.83, found: C 74.08, H 5.42, N 14.71. MS (ESI): 567 [M + H]^+^. Product D4: orange solid, yield: 78%, mp: 202–204 °C; FT-IR (KBr, *ν*/cm^−1^): 3411, 3060, 2951, 2881, 1730, 1640, 1596, 1498, 1279, 1235, 832, 797, 776, 756. ^1^H NMR (400 MHz, DMSO-*d*_6_) *δ*: 8.22 (d, *J* = 2.0 Hz, 1H), 7.95 (dd, *J* = 8.8, 1.2 Hz, 4H), 7.52 (d, *J* = 8.0 Hz, 1H), 7.38–7.25 (m, 8H), 7.03 (tt, *J* = 7.4, 1.0 Hz, 2H), 5.12 (s, 1H), 2.63 (s, 3H), 2.18 (s, 6H). ^13^C NMR (100 MHz, DMSO-*d*_6_) *δ*: 158.2, 150.4, 147.9, 146.8, 146.6, 145.3, 141.4, 137.1, 134.1, 131.6, 130.4, 128.7, 127.0, 123.2, 122.1, 119.4, 115.4, 102.2, 100.3, 56.2, 28.5, 17.5, 13.7. Anal. calcd for C_34_H_28_N_6_O_2_: C 73.90, H 5.11, N 15.21, found: C 74.02, H 5.23, N 15.15. Product D5: yellow solid, yield: 71%, mp: 200–202 °C; FT-IR (KBr, *ν*/cm^−1^): 3412, 2952, 1737, 1640, 1597, 1496, 1279, 1239, 765. ^1^H NMR (400 MHz, DMSO-*d*_6_) *δ*: 8.24 (s, 1H), 7.97 (d, *J* = 8.4 Hz, 4H), 7.54 (d, *J* = 8.0 Hz, 1H), 7.40–7.26 (m, 8H), 7.05 (t, *J* = 7.0 Hz, 2H), 5.15 (s, 1H), 3.88 (s, 3H), 2.64 (s, 3H), 2.21 (s, 6H). ^13^C NMR (100 MHz, DMSO-*d*_6_) *δ*: 158.2, 150.5, 148.0, 147.1, 146.7, 145.2, 141.4, 137.1, 134.2, 131.7, 130.4, 128.7, 127.0, 123.2, 122.2, 119.5, 115.4, 102.2, 100.2, 56.2, 28.4, 17.5, 13.7. Anal. calcd for C_35_H_30_N_6_O_3_: C 72.15, H 5.19, N 14.42, found: C 72.27, H 5.09, N 14.51. Product D6: yellow solid, yield: 76%, mp: 202–203 °C; FT-IR (KBr, *ν*/cm^−1^): 3414, 2956, 1729, 1641, 1596, 1496, 1283, 773. ^1^H NMR (400 MHz, DMSO-*d*_6_) *δ*: 8.20 (s, 1H), 7.96 (d, *J* = 8.0 Hz, 4H), 7.83 (d, *J* = 8.4 Hz, 2H), 7.59 (d, *J* = 8.4 Hz, 2H), 7.32 (t, *J* = 7.6 Hz, 4H), 7.24 (s, 1H), 7.05 (t, *J* = 7.2 Hz, 2H), 5.15 (s, 1H), 3.87 (s, 3H), 2.19 (s, 6H). ^13^C NMR (100 MHz, DMSO-*d*_6_) *δ*: 158.2, 151.1, 148.3, 146.7, 144.5, 141.3, 134.7, 134.2, 129.9, 128.7, 125.8, 124.1, 123.3, 119.5, 118.4, 102.1, 97.1, 56.2, 28.4, 13.7. Anal. calcd for C_34_H_27_ClN_6_O_3_: C 67.71, H 4.51, N 13.94, found: C 67.54, H 5.05, N 13.77. Product D7: yellow solid, yield: 68%, mp: 179–180 °C; FT-IR (KBr, *ν*/cm^−1^): 3412, 3060, 2948, 2881, 1640, 1596, 1499, 1288, 1233, 1058, 797, 772, 693. ^1^H NMR (400 MHz, DMSO-*d*_6_) *δ*: 8.46 (d, *J* = 2.4 Hz, 1H), 7.98 (d, *J* = 7.6 Hz, 4H), 7.80 (d, *J* = 7.2 Hz, 2H), 7.57–7.54 (m, 3H), 7.48 (t, *J* = 7.2 Hz, 1H), 7.34 (t, *J* = 7.8 Hz, 4H), 7.06 (t, *J* = 7.4 Hz, 2H), 6.89 (d, *J* = 8.4 Hz, 1H), 5.11 (s, 1H), 2.22 (s, 6H). ^13^C NMR (100 MHz, DMSO-*d*_6_) *δ*: 158.3, 158.0, 152.7, 146.7, 145.5, 141.3, 134.2, 130.6, 129.8, 128.8, 128.7, 123.3, 122.5, 119.5, 118.7, 15.7, 102.2, 28.5, 13.6. Anal. calcd for C_33_H_26_N_6_O_2_: C 73.59, H 4.87, N 15.60, found: C 73.67, H 4.63, N 15.78. Product D8: yellow solid, yield: 81%, mp: 248–250 °C; FT-IR (KBr, *ν*/cm^−1^): 3411, 2956, 2929, 1733, 1640, 1597, 1499, 1236. ^1^H NMR (400 MHz, DMSO-*d*_6_) *δ*: 8.43 (d, *J* = 2.4 Hz, 1H), 7.95 (d, *J* = 7.6 Hz, 4H), 7.79 (d, *J* = 8.8 Hz, 2H), 7.59 (d, *J* = 8.8 Hz, 2H), 7.53 (dd, *J* = 8.6, 2.4 Hz, 1H), 7.32 (t, *J* = 8.0 Hz, 4H), 7.04 (t, *J* = 7.4 Hz, 2H), 6.87 (d, *J* = 8.6 Hz, 1H), 5.06 (s, 1H), 2.18 (s, 6H). ^13^C NMR (100 MHz, DMSO-*d*_6_) *δ*: 158.3, 151.3, 146.6, 145.4, 141.3, 134.8, 134.2, 129.9, 129.1, 129.0, 128.7, 124.2, 123.3, 119.5, 118.8, 115.7, 102.1, 28.4, 13.6. Anal. calcd for C_33_H_25_ClN_6_O_2_: C 69.17, H 4.40, N 14.67, found: C 69.29, H 4.52, N 14.76. Product D9: yellow solid, yield: 55%, mp: 175–176 °C; FT-IR (KBr, *ν*/cm^−1^): 3410, 2956, 1731, 1639, 1599, 1497, 1242, 776. ^1^H NMR (400 MHz, DMSO-*d*_6_) *δ*: 8.37 (d, *J* = 2.4 Hz, 1H), 7.95 (d, *J* = 8.0 Hz, 4H), 7.77 (d, *J* = 8.8 Hz, 2H), 7.47 (dd, *J* = 8.4, 2.4 Hz, 1H), 7.31 (t, *J* = 8.0 Hz, 4H), 7.08 (d, *J* = 8.8 Hz, 2H), 7.04 (t, *J* = 7.4 Hz, 2H), 6.84 (d, *J* = 8.4 Hz, 1H), 5.06 (s, 1H), 3.85 (s, 3H), 2.18 (s, 6H). ^13^C NMR (100 MHz, DMSO-*d*_6_) *δ*: 161.3, 158.3, 157.2, 146.8, 146.6, 145.5, 141.4, 134.0, 129.4, 129.1, 128.7, 128.3, 124.2, 123.2, 119.4, 114.9, 102.2, 56.0, 28.5, 13.6. Anal. calcd for C_34_H_28_N_6_O_3_: C 71.82, H 4.96, N 14.78, found: C 71.61, H 4.79, N 14.83. Product D10: brown solid, yield: 90%, mp: 234–235 °C; FT-IR (KBr, *ν*/cm^−1^): 3411, 2959, 2929, 2868, 1729, 1640, 1598, 1499, 1340, 1286, 796, 752, 688. ^1^H NMR (400 MHz, DMSO-*d*_6_) *δ*: 8.43 (s, 1H), 8.38 (d, *J* = 8.8 Hz, 2H), 7.97 (d, *J* = 8.8 Hz, 2H), 7.87 (d, *J* = 8.4 Hz, 4H), 7.68 (d, *J* = 8.4 Hz, 1H), 7.39 (t, *J* = 7.6 Hz, 4H), 7.15 (t, *J* = 7.2 Hz, 2H), 6.97 (d, *J* = 8.6 Hz, 1H), 5.16 (s, 1H), 2.27 (s, 6H). ^13^C NMR (100 MHz, DMSO-*d*_6_) *δ*: 159.6, 158.2, 156.1, 148.0, 146.7, 145.7, 139.7, 132.7, 129.1, 128.7, 125.5, 124.6, 123.4, 120.7, 120.2, 116.2, 103.0, 28.3, 13.0. Anal. calcd for C_33_H_25_N_7_O_4_: C 67.92, H 4.32, N 16.80, found: C 70.11, H 4.25, N 16.96. MS (ESI): 584 [M + H]^+^.

## 3. Result and discussion

### 3.1 Synthesis of the desired dyes D1–D10

**Scheme 1:**
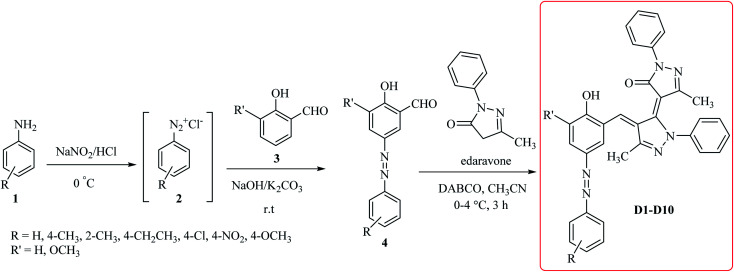
General method for the efficient synthesis of the dyes D1–D10.

The starting material the azo compounds 4 is readily prepared from the commercial available aniline derivatives 1. Diazotization of 1 with NaNO_2_/HCl at 0–4 °C give its corresponding diazonium salt 2. Aza coupling of 2 using 2-hydroxybenzaldehyde 3 under basic condition at room temperature affords the corresponding azo compounds 4 in high yield (55–90%) and excellent purity. With the azo compounds 4 in hand, attention was focused on the synthesis of desired dyes D1–D10 by using the condensation reaction of 4 with edaravone in the presence of DABCO as a catalyst (Scheme 1).

A diverse range of azo derivatives having electron-donating as well as electron-withdrawing substituents reacted with edaravone successfully and the corresponding dyes were obtained in excellent yields. The results are summarized in Table 1. In general, electron-withdrawing substituents furnished the desired products in excellent yields (Table 1, D2 and D10).

**Table 1:** Synthesis of the desired dyes D1–D10

Molecule	Mp (°C)	Yield^a^ (%)	Molecule	Mp (°C)	Yield (%)
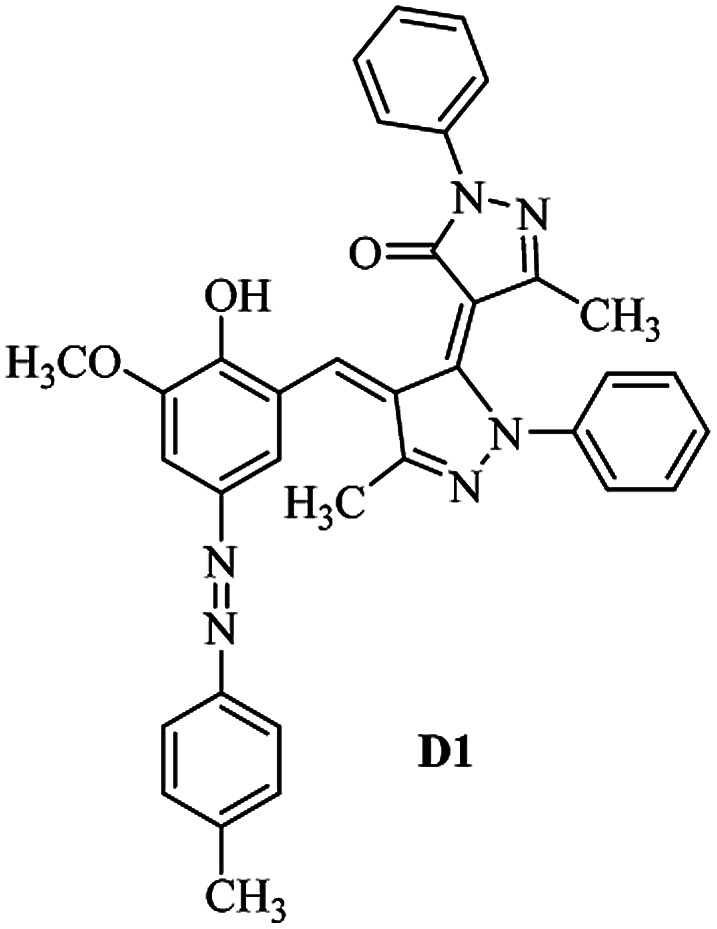	204	77	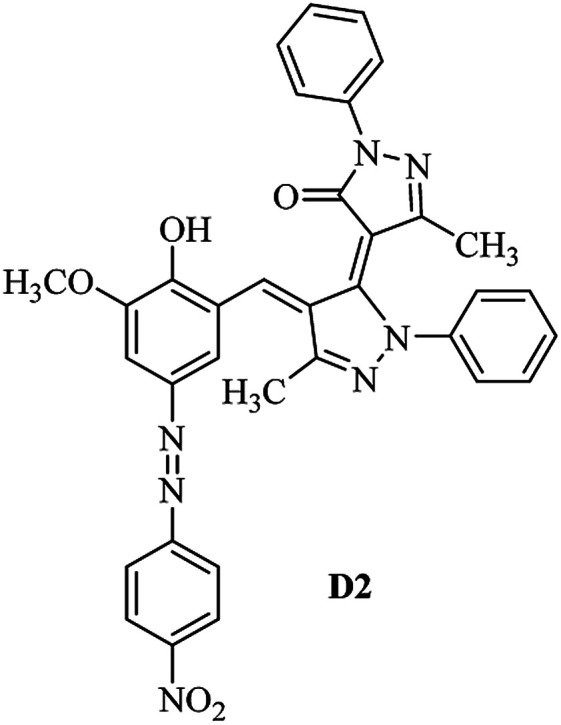	176	87
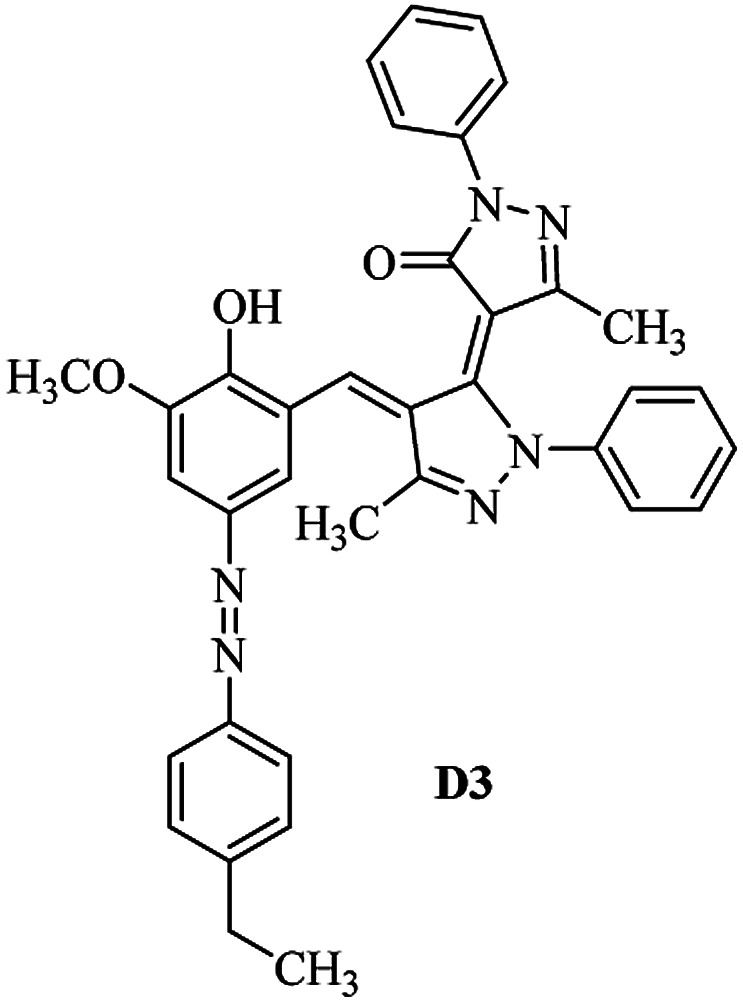	177	80	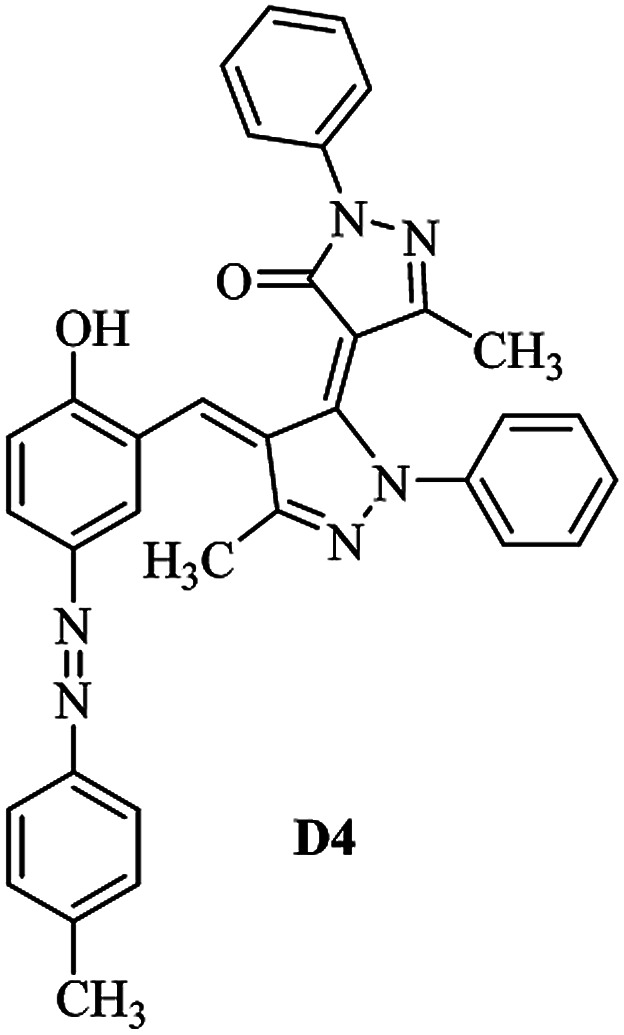	202	78
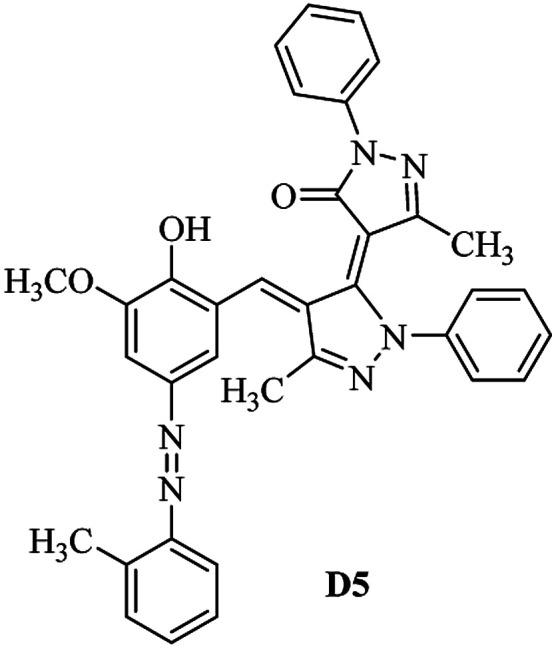	202	71	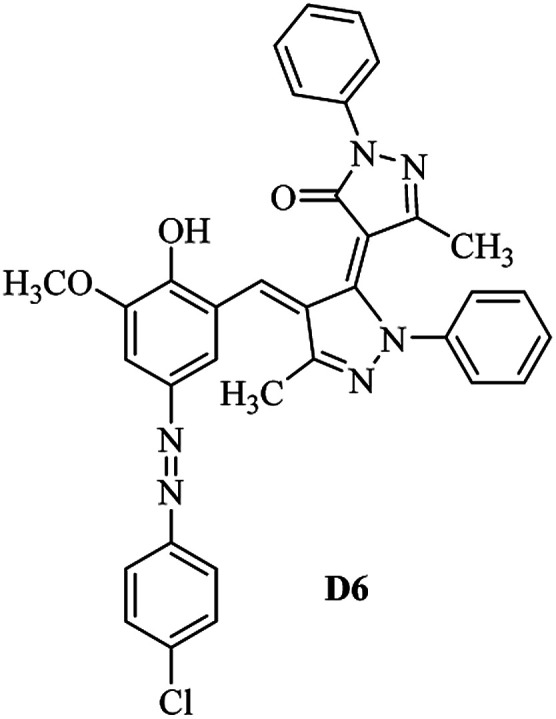	203	76
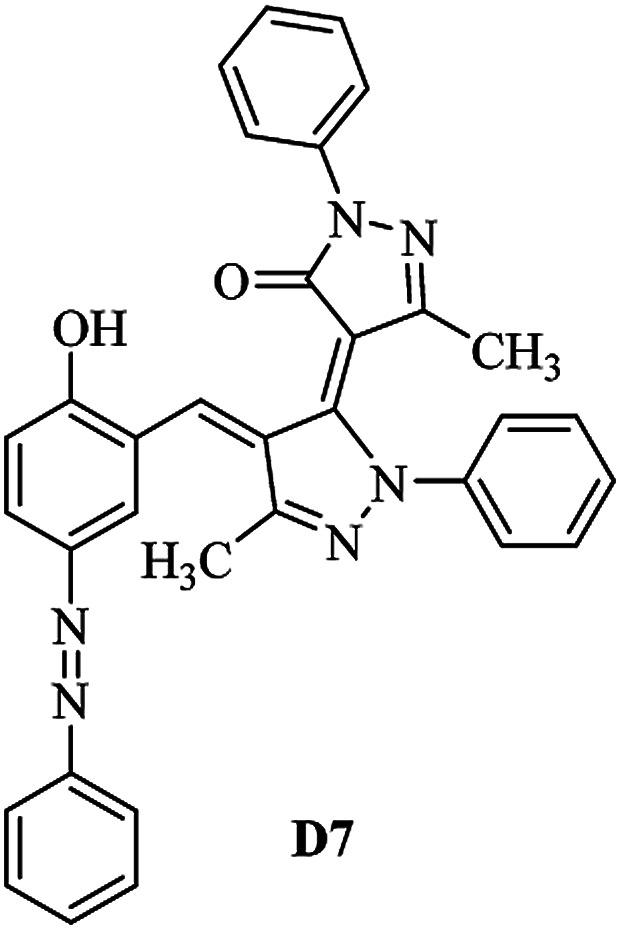	180	68	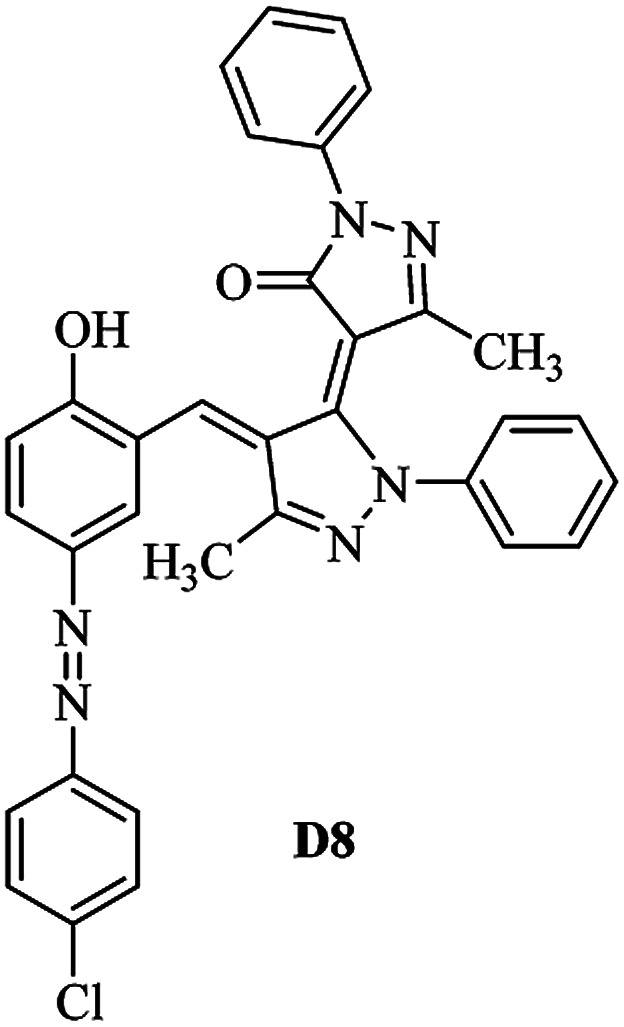	248	81
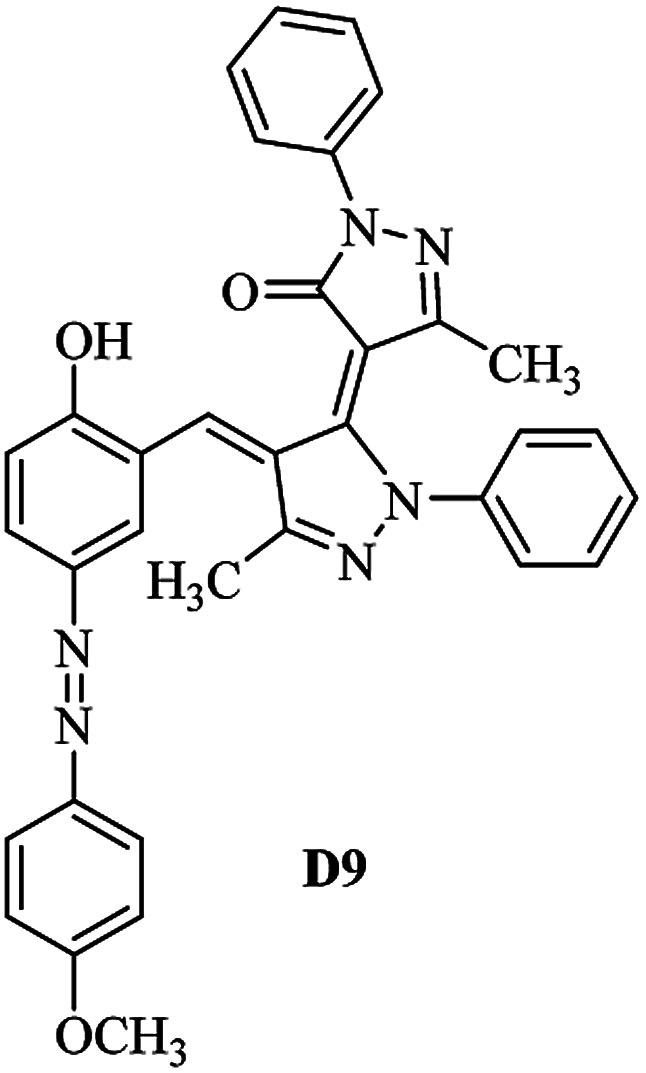	175	55	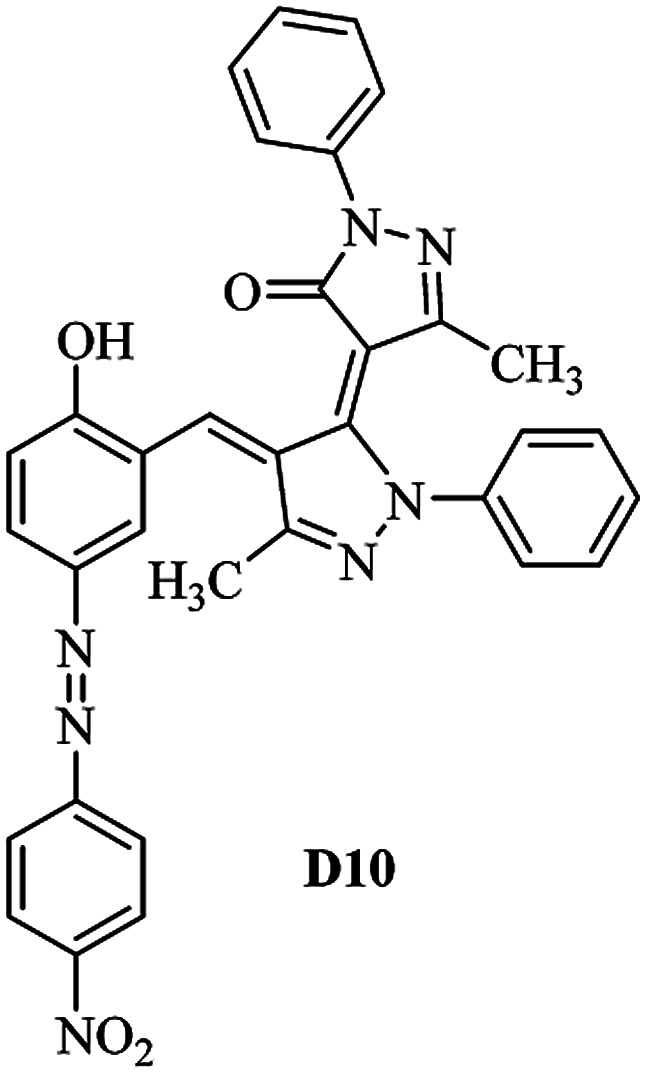	234	90

a Yields after work-up.

The FT-IR, ^1^H NMR, ^13^C NMR, UV-vis and CHN data of the products clearly indicated the formation of the desired products D1–D10 (Experimental section and ESI†). As a representative example FT-IR, ^1^H NMR and ^13^C NMR data of D1 are discussed. FT-IR spectrum of D1 revealed a broad band around 3411 cm^−1^, corresponding to adsorption band of the O–H group. Characteristic peaks at 1729 and 1641 cm^−1^ can be related to C=O and C=N bonds, respectively. The ^1^H NMR spectrum of D1 consists of two peaks (*δ* 2.18 and 2.39 ppm) for three methyl groups, a singlet for the O–CH_3_ (*δ* 3.86 ppm), a characteristic singlet for the vinylic proton (*δ* 3.86 ppm) and 16 aromatic hydrogens at *δ* = 7.01–8.15 ppm. The ^1^H-decoupled ^13^C NMR spectrum of D1 showed the presence of resonances at *δ* = 13.7, 21.4, 28.4 and 56.2 ppm readily recognized as the methyl carbons, resonances at *δ* = 97.2–150.6 ppm for alkene and aromatic carbons and a resonance at *δ* = 158.2 ppm for carbonyl carbon.

**Scheme 2:**
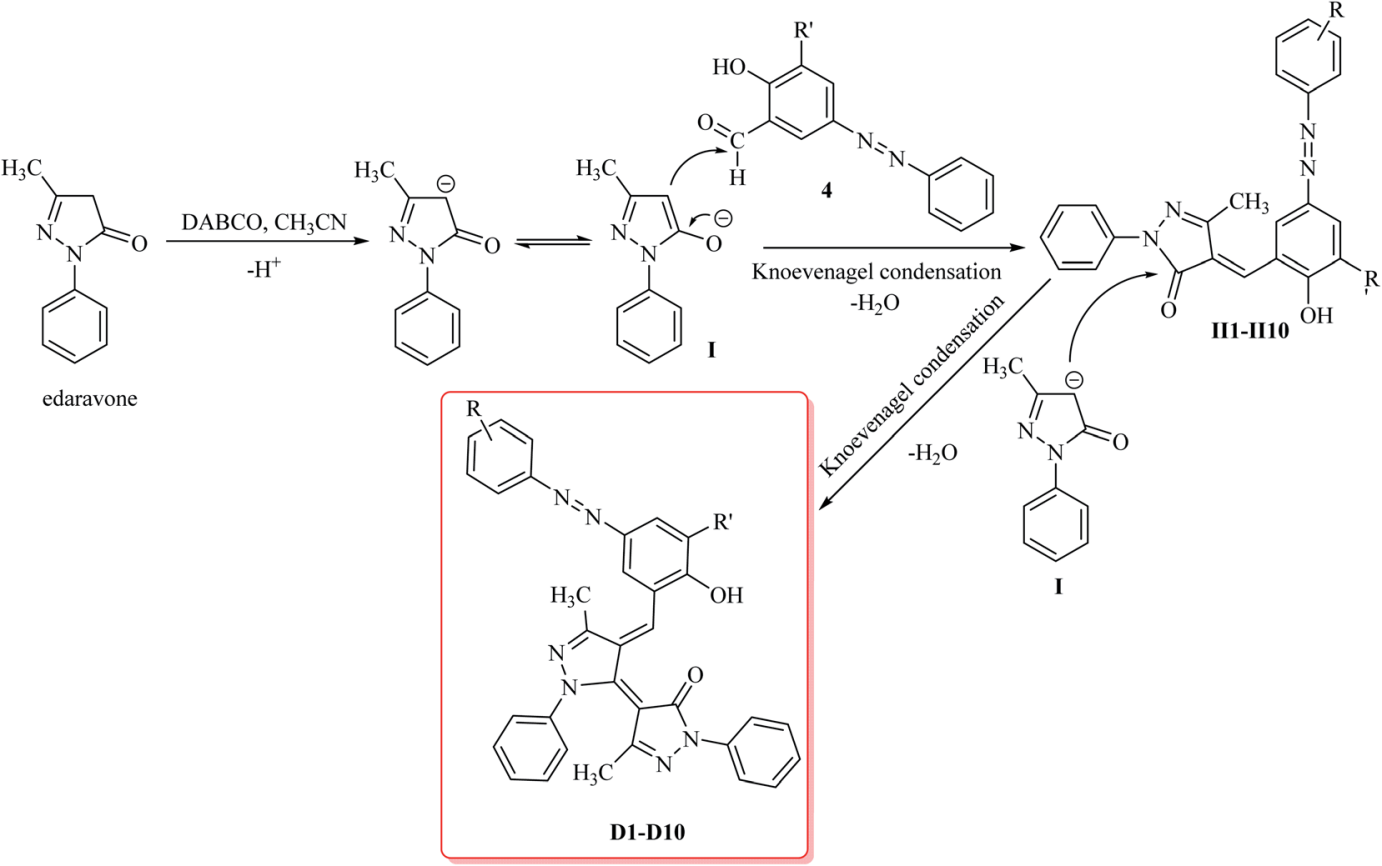
Proposed mechanism for the formation of novel edaravone-based azo dyes D1–D10.

Mechanistically, it is conceivable that the reaction involves the initial deprotonation of edaravone as a hydrogen active compound by DABCO. Then, the carbaldehyde functional group of azo compounds 4 undergoes the Knoevenagel condensation by the enolate ion of edaravone I affording to intermediate II. Subsequent, another the Knoevenagel condensation between intermediate II and the enolate ion I leads to formation of the novel edaravone-based azo dyes D1–D10 with high yields (Scheme 2).

### 3.2 The UV-visible spectra and solvatochromic studies of four synthesized dyes (D1–D4)

We recorded the absorption spectra of D1–D4 in eight solvents with different polarity at a concentration of 3 × 10^−5^ M in the range of 300–700 nm to study of the solvent effects on spectral features of the synthesized dyes (Table 2). In Table 2, the solvents are arranged in the order of increasing polarity. Refractive index (*n*), dielectric constant (*ε*) and the solvatochromic parameters including hydrogen bond donating ability (*α*), hydrogen bond accepting ability (*β*) and dipolarity/polarizability polarity scale (π*) were taken from the literature.^43–45^

**Table 2:** Experimental electronic absorption maxima for investigated dyes D1–D4 and solvent parameters

Solvents	π*	*α*	*β*	*ε*	*n*	*λ*_max_ (nm)			
						D1	D2	D3	D4
Ethyl acetate	0.54	0.00	0.45	6.68	1.3723	367	423	365	355
Ethanol	0.54	0.83	0.77	25.33	1.3611	375	462	360	355
Chloroform	0.58	0.44	0.00	4.81	1.4459	382	450	358	364
Methanol	0.6	0.93	0.62	33.10	1.3288	372	452	360	369
Acetone	0.71	0.08	0.48	21.01	1.3588	377	453	362	365
Acetonitrile	0.75	0.19	0.31	37.50	1.3442	367	447	352	357
DMF	0.87	0.00	0.69	38.25	1.4305	387	494	391	429
DMSO	1	0.00	0.76	47.24	1.4770	377	467	375	384

As can be seen from Table 2, the maximum absorption band (*λ*_max_) in the range of 350–500 nm for the studied dyes D1–D5 can be attributed to n → π* and/or π → π* electronic transitions of azo chromophores.^46^

**Fig. 1:**
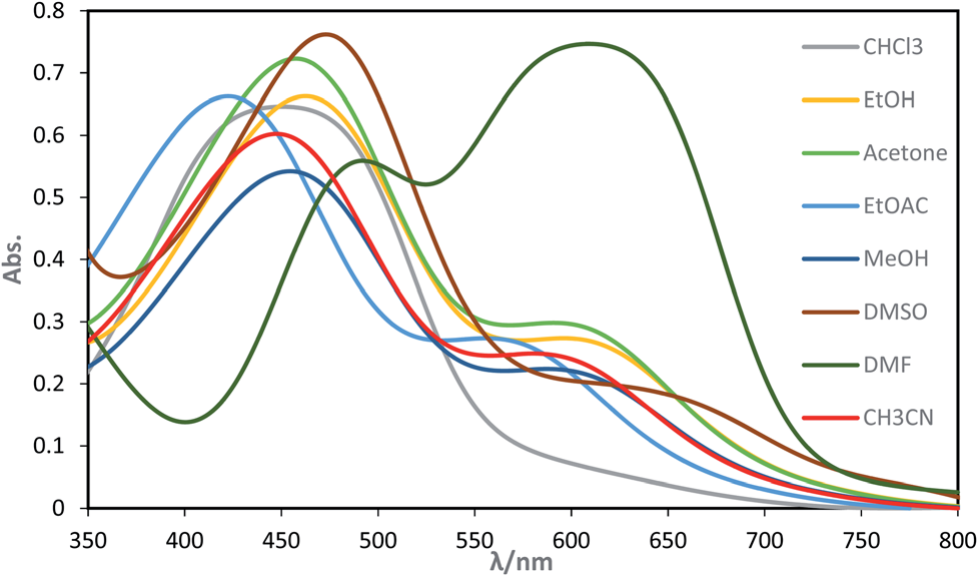
Electronic absorption spectra of dye D2 in different solvents.

The dyes displayed two absorption peaks in most of the solvents within the range of 350–750 nm (Fig. 1). The first one which is located at the range of 350–550 could be attributed to the azo forms (raised due to light absorption by azo form of the dyes) of the synthesized dyes and the second one which is appeared within the range of 550–700 nm could be attributed to the hydrazo form (raised due to light absorption by hydrazo form of the dyes) of the obtained dyes.

**Scheme 3:**
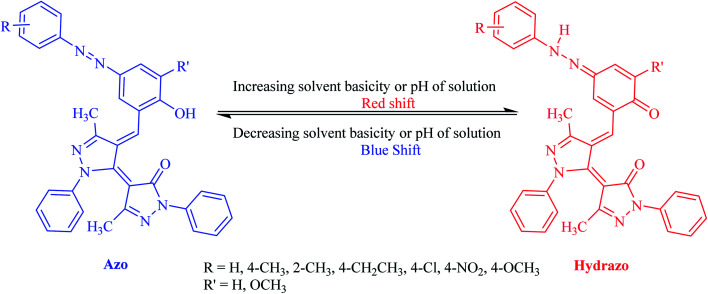
Azo–hydrazo equilibrium for synthesized dyes.

The equilibrium between azo and hydrazo forms of the dyes is represented on Scheme 3. The both observed absorption peaks of the dyes showed strong solvent dependency, denoting bathochromic effect (positive solvatochromism) which are influenced with the solvents polarity.

Intensity of the peaks for both azo and hydrazo forms of the dyes are not equal and the whose for azo form has higher intensity than that of hydrazo one at most of the solvents, except DMF. In addition, the intensity of the both azo and hydrazo absorption peaks are different at the various solvents indicating that the concentration of the azo and hydrazo forms of the dyes are not equal at various solvents and are depended on the solvent nature specially its basicity as will be discussed below. Higher peak intensity for the first absorption which is attributed to azo form of the dyes indicates that the azo form is the dominant form of the synthesized dyes at most of the solvents. It's worth to not that the both of the absorption peaks are shifted in consistent with the change in solvent polarity.

Basicity of the solvents could affect the equilibrium between azo and hydrazo forms of the dyes in a way that as the basicity of the solvent increased, the equilibrium between azo and hydrazo forms of the dyes is shifted to the hydrazo form. Therefore hydrazo forms are the dominant forms in the basic solutions. Hydrazo forms absorb the light at the higher wavelengths because of the higher electron resonance capability and having longer resonance system. This is occurred because of the deprotonation of the phenolic hydroxyl group by the solvent molecules that leads to the enhancing of the electron resonance and then shifting of the absorption peaks to the higher wavelengths (red shift).The spectral shift is mainly the result of solute–solvent interactions which are led to a better stabilization of the π* antibonding orbital in comparison to the π bonding orbital in polar solvents. It is well known that the solvent nature affects the position and shape of the absorption spectra. Solvatochromic parameters such as (*n* and *ε*) and (π*, *α* and *β*) were used to evaluation of the changes which are appeared in the absorption peaks of the dyes due to the solvent change. Investigations of the data in Table 2 indicated that there is regular change between the *λ*_max_ values of the dyes and solvatochromic parameters (π*, *ε* and *β*) values of the studied solvents. On the other hand, all studied dyes, exhibit a bathochromic shift (red shift) as a result of an increase in the solvent polarity scale (π*) and hydrogen bond accepting ability (*β*).

### 3.3 Substituent effects

Absorption maxima of azobenzene dyes are strongly influenced by the substituents in the phenyl ring especially at *para*-position.^47^ Electron donor and electron acceptor substituents decreased and increased the donor–acceptor polarization of the dye molecules, respectively and leads to lengthening and shortening of the resonance system respectively. Increasing and decreasing of the donor–acceptor polarization capacity of the dye molecules also depends on the polarity of the solvent molecules. Increasing and decreasing of the absorption maxima of a dye molecule due to change in solvent polarity are known as bathochromic and hypsochromic shifts respectively. Dye D2 and dye D1 have nitro and methyl substituents at the *para* position of their phenyl ring, therefore it could be expected that the donor–acceptor polarization in the first one is higher than that in the second one and then dye D2 absorption maxima appears at higher wavelength than the dye D1 as verified by the results of the Table 2. As listed in Table 2, the *λ*_max_ for dye D2 (azo form, 447 nm) is about 80 nm higher than that of dye D1 (azo form, 367 nm) in acetonitrile as solvent. The nearly same results are obtained for difference between azo form *λ*_max_ values of dye D2 and dye D1 at another solvents. For all dyes (D1–D4) bathochromic shift appeared with the increasing of the solvents polarity. Bathochromic shift values are higher for *para* electron acceptor substituted dyes than *para* electron donor substituted ones.

### 3.4 Azo–hydrazo tautomers at different pH values

**Scheme 4:**
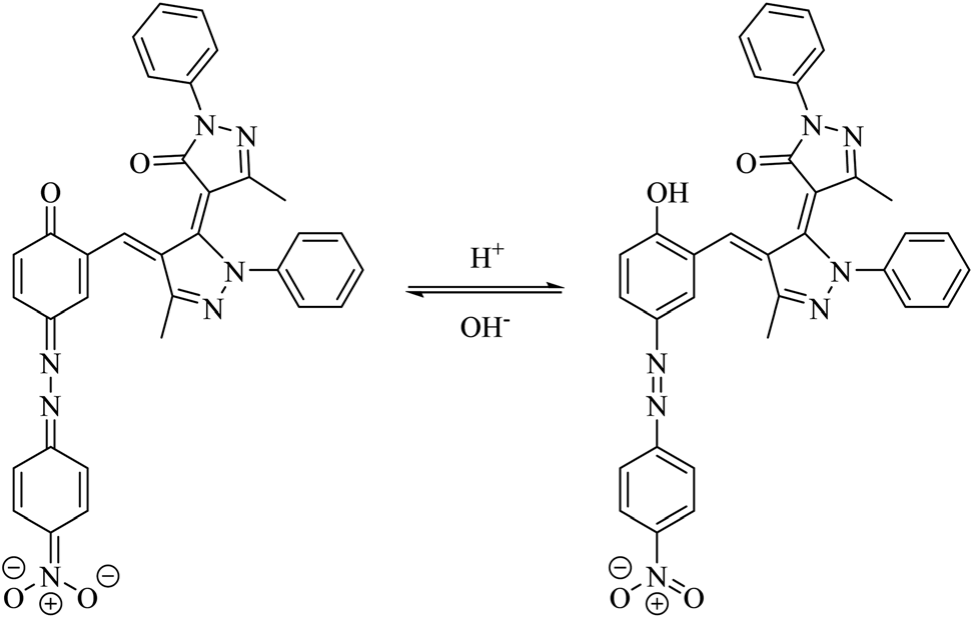
Acid–base equilibrium of the dye D10.

**Fig. 2:**
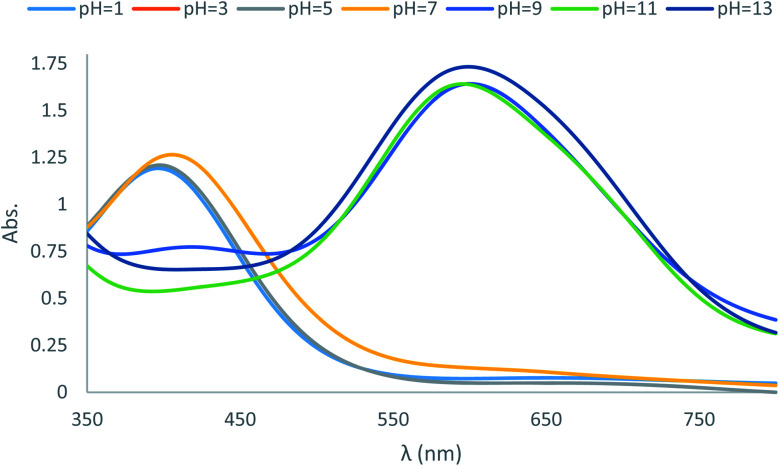
Absorption spectra of the dye D10 in acidic and basic solutions.

In the next part of the investigation, the pH values of the synthesized dyes were also determined spectrophotometrically in 80% (v/v) DMSO–H_2_O mixtures (aqueous hydrochloric acid and sodium hydroxide) at 25 ± 2 °C. A digital pH meter Genway model 3505 was employed for determination of pH. The instrument was accurate to ±0.01 pH unit (Table 3). Comparison of the absorption maxima of the dyes revealed a red shift in *λ*_max_ of the main visible band in basic solutions. This shift can be explained on the basis that the dyes especially dye D10 with acceptor substituent groups exist in acid–base equilibrium (Scheme 4). Considering the *λ*_max_ in Table 3, these dyes in basic solutions shifts to higher wavelengths, which are probably related to the prominence of hydrazone forms in basic medium, while in neutral and acidic solutions the dyes exists in azo forms. The UV-vis spectroscopic study on dye D10 clearly indicates that the present dye exists in azo form in solutions with pH = 1, 3, 5 and 7, in hydrazo form in solutions with pH = 11 and 13 and in acid–base equilibrium forms in solution with pH = 9 as shown in Fig. 2.

**Table 3:** Electronic absorption spectral data of compounds D1, D2, D3, D7 and D10 in acidic and basic solution

*λ*_max_ (nm)							
Dye no.	pH = 1	pH = 3	pH = 5	pH = 7	pH = 9	pH = 11	pH = 13
D1	373	374	374	380	523	501	500
D2	449	451	452	449	624	623	614
D3	360	360	360	361	495	480	476
D7	360	360	360	361	495	480	476
D10	396	397	397	405	600	595	599

### 3.5 DFT calculation studies

#### 3.5.1 Geometry and stability

**Fig. 3:**
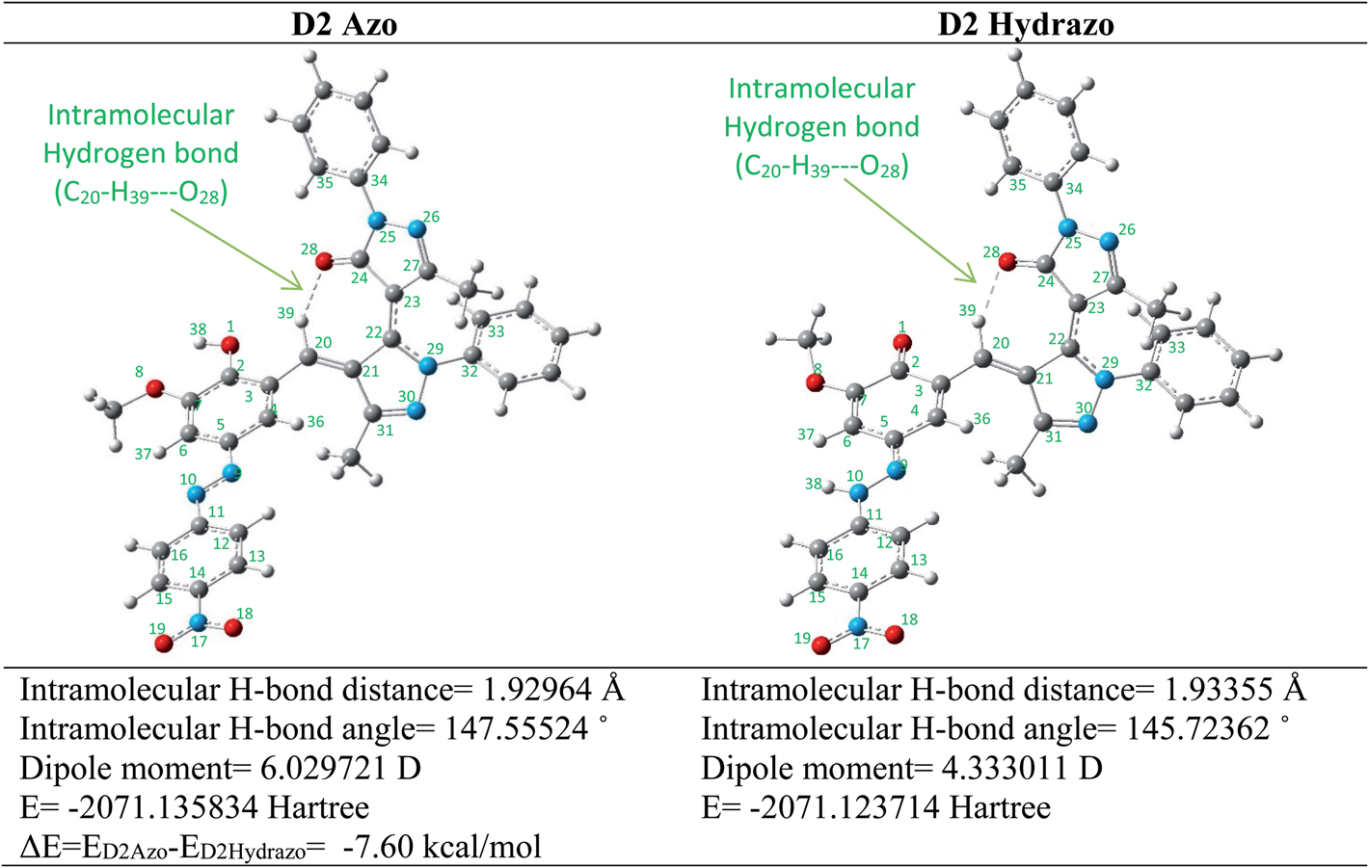
Optimized structures of the dye D2Azo and D2Hydrazo tautomers at B3LYP/6-31+G(d,p) level.

To better understand the geometrical characteristic and stability of the synthesized dyes in both azo and hydrazo forms, Density Functional Theory (DFT) calculations at the B3LYP/6-31+G(d,p) level were used.^40,41,48^ It's worth noting that for all of the optimized structures, the frequency calculations also were carried out to ensure that the obtained geometries are true minima. Two possible tautomers for dye D2 were designed and their initial structures were optimized using B3LYP/6-31+G(d,p) level of theory (Fig. 3).

As shown in Fig. 3 the most stable geometry for azo and hydrazo tautomers of the dye D2 are located at the left corner of the Fig. 3 with the electronic energy of −2071.135834 hartree and at the right corner of Fig. 3 with the electronic energy of −2071.123714 hartree, respectively. The energy difference between these two tautomers are calculated and located below of the Fig. 3. As revealed from the results of energy calculations the D2Azo tautomer is about 7.60 kcal mol^−1^ more stable than D2Hydrazo one. Some calculated geometrical parameters of azo and hydrazo tautomers of the dye D2 were collected in Table 4. As revealed from these data there is some difference in geometrical characteristics of both of the tautomers especially at C11–N10–N9 and N10–N9–C5 angles, C2–O1 and N9–N10 bond distances and C4–C3–C20–C21 dihedral angle.

**Table 4:** Some calculated geometrical data of azo and hydrazo tautomers of the dye D2 using B3LYP/6-31+G(d,p) level of theory

	D2Azo	D2Hydrazo
**Bond distance (Å)**		
C2–O1	1.34721	1.23048
N9–N10	1.26209	1.32508
C5–N9	1.40643	1.32375
N10–C11	1.41751	1.39310
O1(N10)–H38	0.97525	1.01804
C14–N17	1.47140	1.46364
C20H39	1.08938	1.08893
C24–O28	1.24167	1.24040
C3–C20	1.45738	1.45467
C20–C21	1.37097	1.37165

**Bond angle (°)**		
C11–N10–N9	114.78515	121.92451
N10–N9–C5	115.58530	119.94854
C3–C20–C39	116.18416	116.03474
C20–C21–C22	126.84005	127.30365
C22–C23–C24	125.68406	125.68801
N25–C24–O28	125.53320	125.53610
C20–H39–O28	147.55524	145.72362
H39–O28–C24	108.50386	110.39433

**Dihedral angle (°)**		
C11–N10–N9–C5	−179.85988	−179.73656
C12–C11–N10–N9	1.40437	0.75618
N10–N9–C5–C6	0.86567	1.48900
C4–C3–C20–C21	41.10489	42.50262
C22–C23–C24–O28	−2.95999	−3.25010
C27–C23–C22–N29	−26.46981	−25.36452
C22–N29–C32–C33	−31.30726	−30.51262
C24–N25–C34–C35	−4.54651	−5.10406

As revealed from the optimized structures of the both D2Azo and D2Hydrazo tautomers there is an intramolecular hydrogen bond in theirs optimized structure. Intramolecular hydrogen bond formed between oxygen atom of carbonyl functional group as hydrogen bond acceptor and CH of newly formed C=C double bond as hydrogen bond donor and give them additional stability. Intramolecular hydrogen bond in D2Azo is stronger than that of D2Hydrazo tautomer. This matter concluded by the shorter hydrogen bond distance and linear hydrogen bond angle of D2Azo (1.930 Å, 147.6°) in comparison with D2Hydrazo hydrogen bond distance and angle (1.934 Å, 145.7°).

#### 3.5.2 UV-visible spectra prediction

In this part, we get help chemical computational methods to get information about UV-visible absorption capability of the products. Therefore, optimized ground state geometry of the azo and hydrazo forms of the D2 have submitted to calculation of the UV-visible spectra using time-dependent (TD) DFT method at B3LYP/6-31+G(d,p) computational level in both gas and solution phase. Time-Dependent Density Functional Theory (TD-DFT)^49^ has recently emerged as a powerful method for investigating the static and dynamic properties of the organic molecule in its excited states, allowing for the best compromise between accuracy and computational cost.^50^ The calculated absorption maxima (*λ*_max_) that is a function of the electron availability for the D2 in both tautomeric forms and their corresponding oscillator strength are listed in Table 5.

**Table 5:** Calculated absorption maxima (*λ*_max_) and corresponding oscillator strength of the D2Azo and D2Hydrazo

D2Azo				D2Hydrazo			
Gas		DMSO		Gas		DMSO	
*λ*/nm	*f*	*λ*/nm	*f*	*λ*/nm	*f*	*λ*/nm	*f*
423	0.2790	458	0.7756	475	0.5292	533	0.4351

**Fig. 4:**
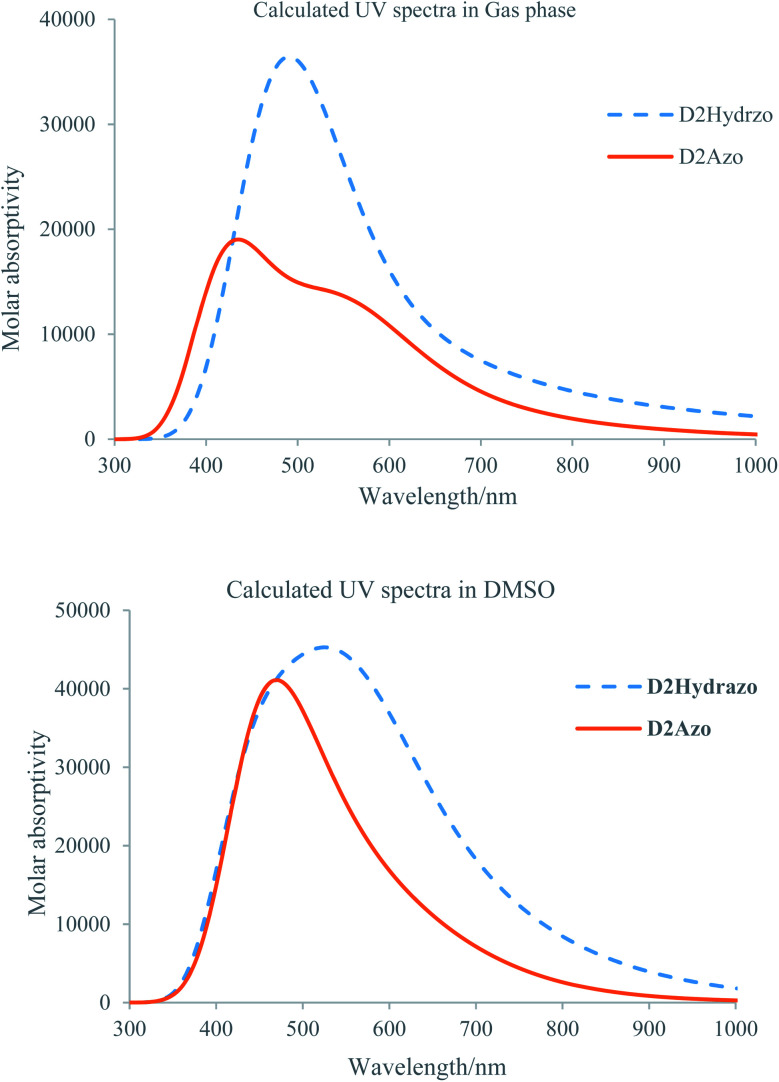
Calculated UV-visible spectra for the D2Azo and D2Hydrazo in gas (upper plot) and DMSO (lower plot).

Fig. 4 shows the resulted absorption spectra for each types of the D2 tautomers over the wavelength range of 200–800 nm in both gas and solution phase which are obtained using above-mentioned calculation methods. Analysis of the obtained plots for D2 tautomers and compare of them with the corresponding experimental data from the absorption maxima (*λ*_max_) and intensity points of view clear the following results:

(1) maximum absorption band for D2Azo appeared at 423 and 548 nm in gas and solution phase respectively.

(2) Maximum absorption band for D2Hydrazo appeared at 475 and 533 nm in gas and solution phase respectively.

(3) Maximum absorption band for D2Azo in comparison to the absorption band of D2Hydrazo appeared in lower wavelength in both gas and DMSO nm which is in consistent with the experimentally obtained results.

(4) Calculated UV-visible spectrum for each product at solution phase has higher intensity than that of its gas phase one.

(5) The appeared maximum absorption band for D2Azo and D2Hydrazo dyes correspond to the electronic transitions among the aromatic rings which are attached each other through diazo bond and mainly derived from the contribution of π–π* bands.

(6) The higher maximum absorption band for D2Hydrazo in comparison to D2Azo could be explained using the intensity of π electron resonances among the conjugated system which is higher in D2Hydrazo than D2Azo because of sharing of the phenolic oxygen nonbonding electrons in resonance.

(7) Calculated maximum absorption wavelength (458 nm) for the dye D2 is in good consistency with its experimental maximum absorption wavelength (467 nm).

### 3.6 Antibacterial activity of the synthesized dyes

The antibacterial activities of some synthesized compounds (D1, D2, D3, D7 and D10) were evaluated using the disk diffusion method. The results of antibacterial activity of D1, D2, D3, D7 and D10 are summarized in Table 6. The results revealed different activity of the selected synthesized dyes for antibacterial test against selected Gram positive and Gram negative bacteria for antibacterial activity evaluation. Among tested dyes the D3 and D10 ones don't show any antibacterial activity against both selected Gram positive and Gram negative bacteria while the D1, D2 and D7 dyes are shown appropriate response against to the antibacterial test. The results obtained during the antibacterial test revealed that the D1, D2 and D7 dyes showed selective antibacterial activity against to the Gram negative, Gram negative and Gram positive bacteria respectively as could be seen from Table 6. For antibacterial comparison, the data for ampicillin were also inserted to Table 6.

**Table 6:** Antibacterial activity of compounds D1, D2, D3, D7 and D10 (2 μg μL^−1^ in DMSO)

Dyes	Zone of inhibition in mm									
	Gram negative (*E. coli*)					Gram positive (*S. aureus*)				
	Test 1	Test 2	Test 3	Test 4	Test 5	Test 1	Test 2	Test 3	Test 4	Test 5
D10	—	—	—	—	—	—	—	—	—	—
D3	—	—	—	—	—	—	—	—	—	—
D1	10	9	9	10	10	—	—	—	—	—
D2	12	10	11	12	11	—	—	—	—	—
D7	—	—	—	—	—	12	12	12	13	12
Ampicillin	7					22				

## 4. Conclusion

In summary, ten novel edaravone-based azo dyes were synthesized *via* efficient Knoevenagel condensations of edaravone with azo-coupled *o*-vanillin and salicylaldehyde precursors. This method offers several advantages such as high yields and starts from easily accessible starting materials, which makes it a useful and attractive protocol for the synthesis of edaravone-based azo dyes in a single step operation. The absorption spectra of four selected compounds of dyes were measured in eight solvents having different physical–chemical properties. Bathochromic shift (positive solvatochromism) of these compounds appeared with the increasing of the solvents polarity. Change at equilibrium contents of the azo and hydrazo forms of the dyes at various solvents also explored and the results showed that the hydrazo form is the dominant one at basic solvents while this matter is reverse for the azo form. The sensory feature of the synthesized dyes to the various anions/cations in different matrices is in progress. During computational study some of the important structural futures of the synthesized dyes such as intramolecular hydrogen bond formation is distinguished. The pH test showed that the major tautomer in acidic and basic solutions are azo and hydrazo tautomers respectively. In addition antibacterial test showed that, some of the synthesized dyes could be used selectively against to the only Gram positive in side of the Gram negative ones or *vice versa*.

## Conflicts of interest

There are no conflicts to declare.

## Supplementary Material

RA-010-D0RA06934E-s001
