# Supplementary material for: Novel edaravone-based azo dyes: efficient synthesis, characterization, antibacterial activity, DFT calculations and comprehensive investigation of the solvent effect on the absorption spectra
Source: RSC Adv. 2020 Sep 29;10(59):35729–39. doi: 10.1039/d0ra06934e (PMC9056902; doi:10.1039/d0ra06934e)
Supplement: RA-010-D0RA06934E-s001 [file RA-010-D0RA06934E-s001.pdf]

Electronic Supplementary Material (ESI) for RSC Advances.
This journal is © The Royal Society of Chemistry 2020

Novel edaravone-based azo dyes: Efficient synthesis, characterization, antibacterial activity, DFT calculations and comprehensive investigation of the solvent effect on the absorption spectra

Mohammad Amin Davasaz Rabbani, Behzad Khalili, Hamid Saeidian

^{1}H NMR, and ^{13}C NMR spectra of Novel edaravone-based azo dyes D1-D10

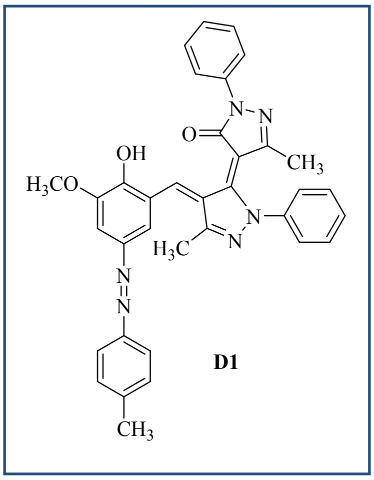

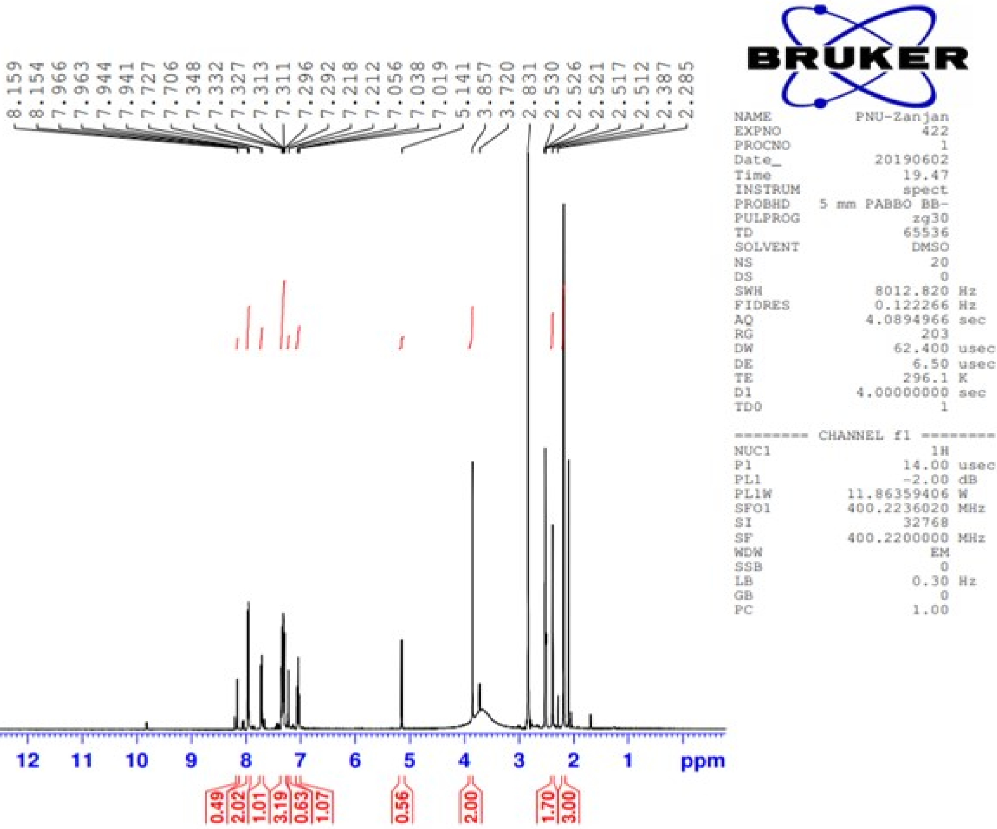

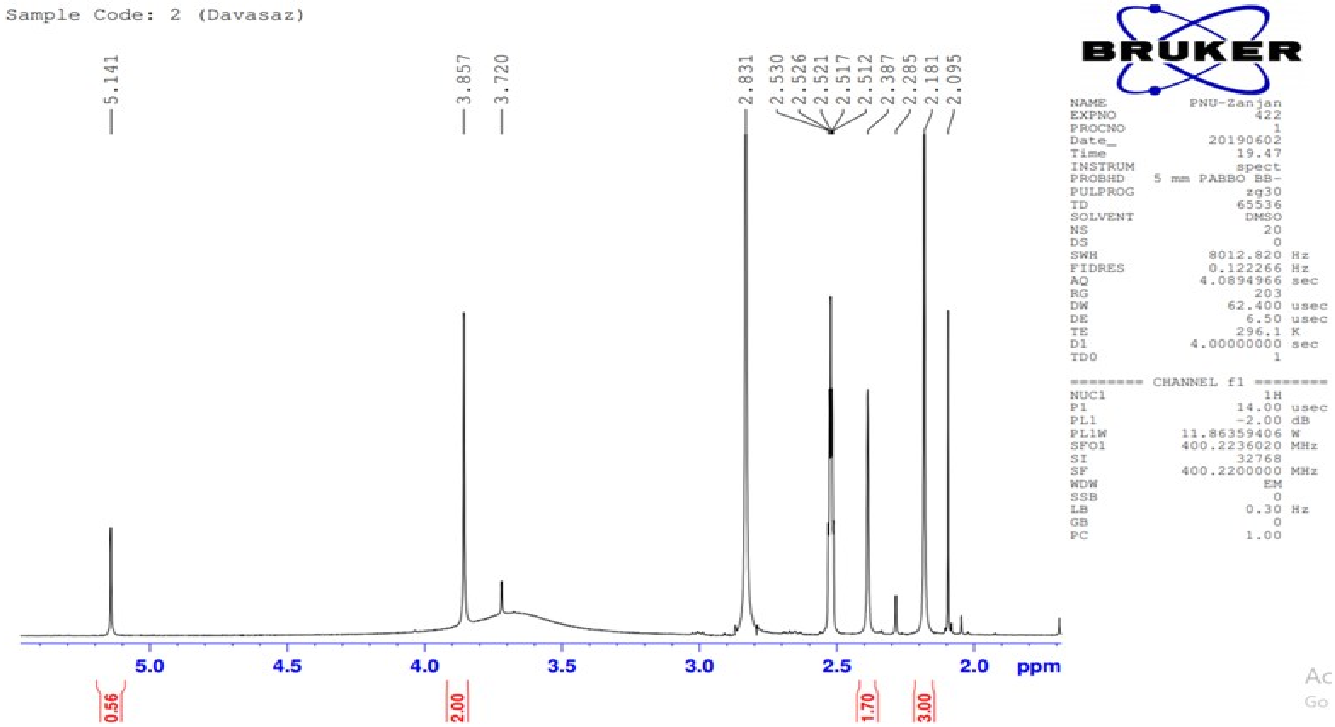

Fig ^{1}H NMR of **D1**

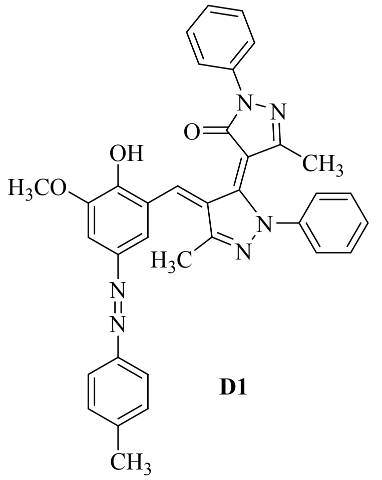

D1

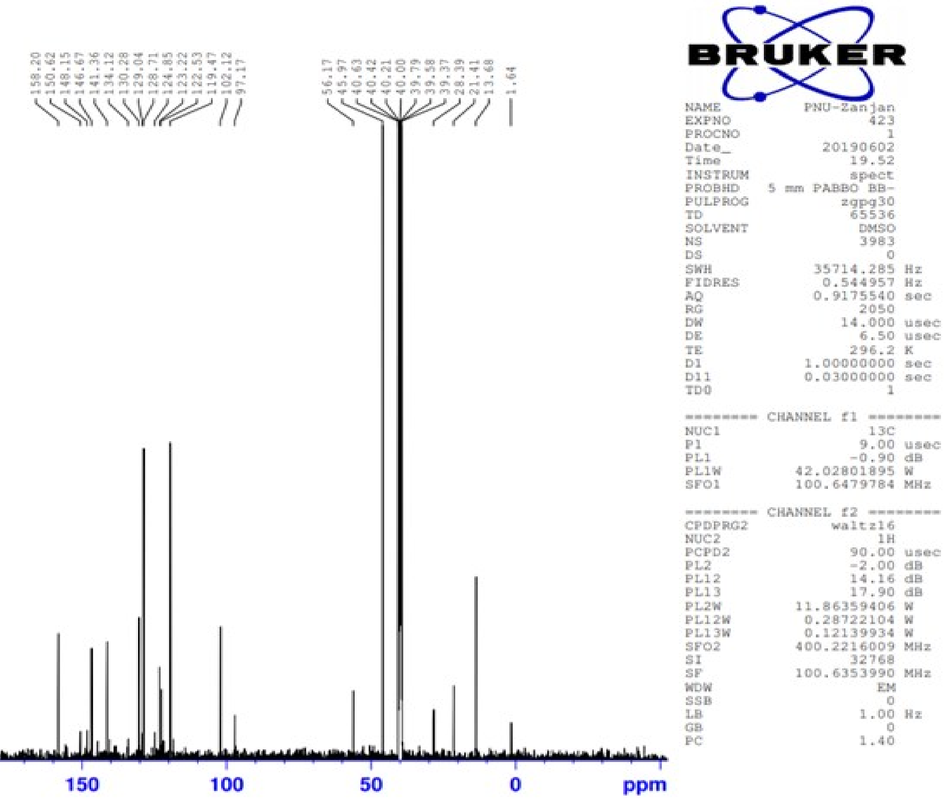

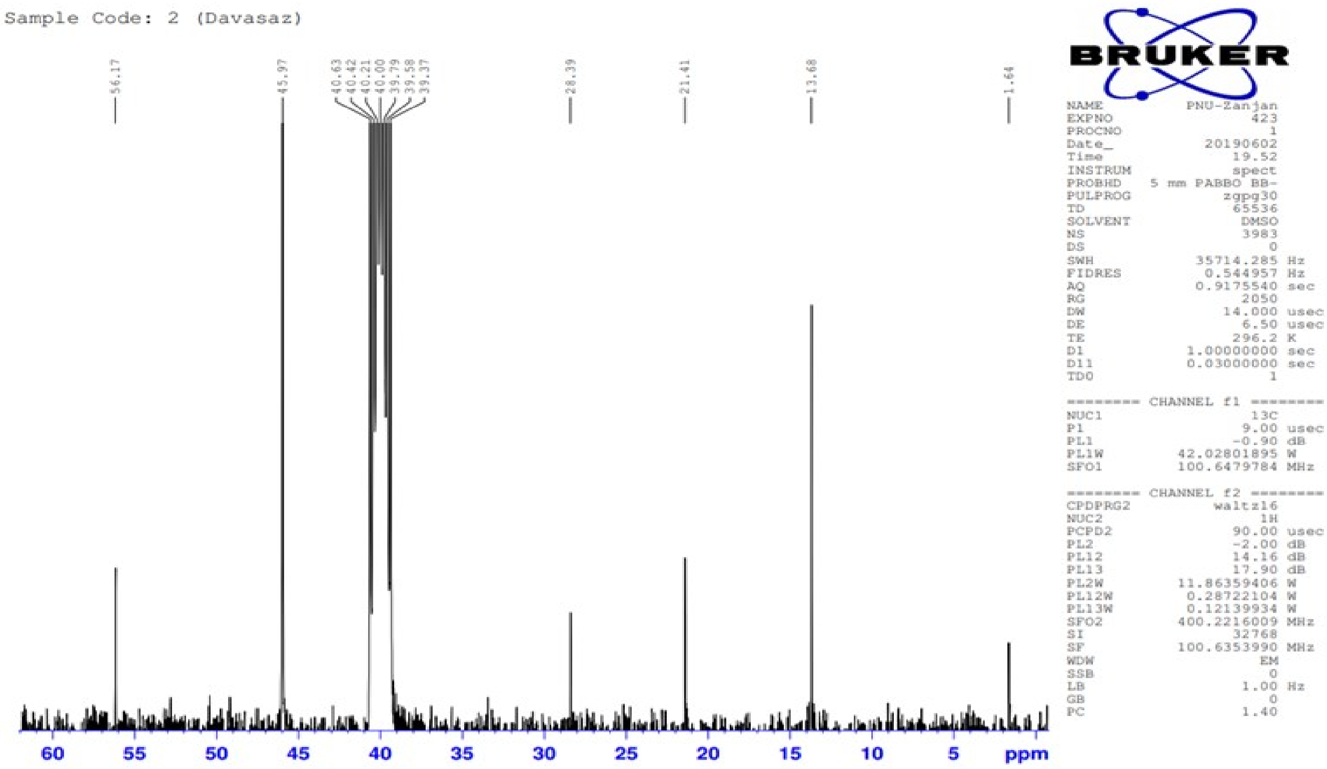

Fig ^{13}C NMR of **D1**.

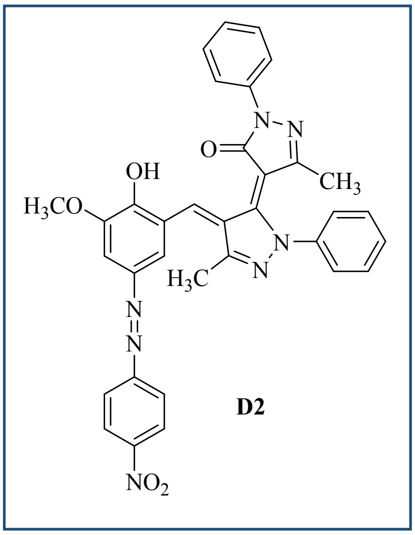

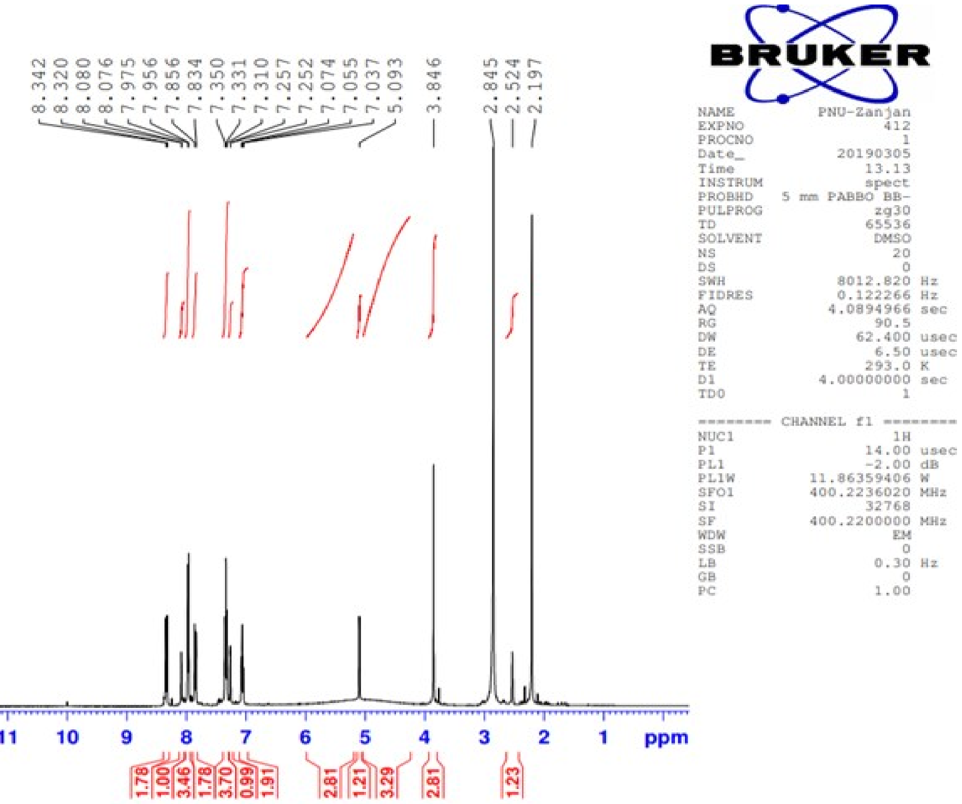

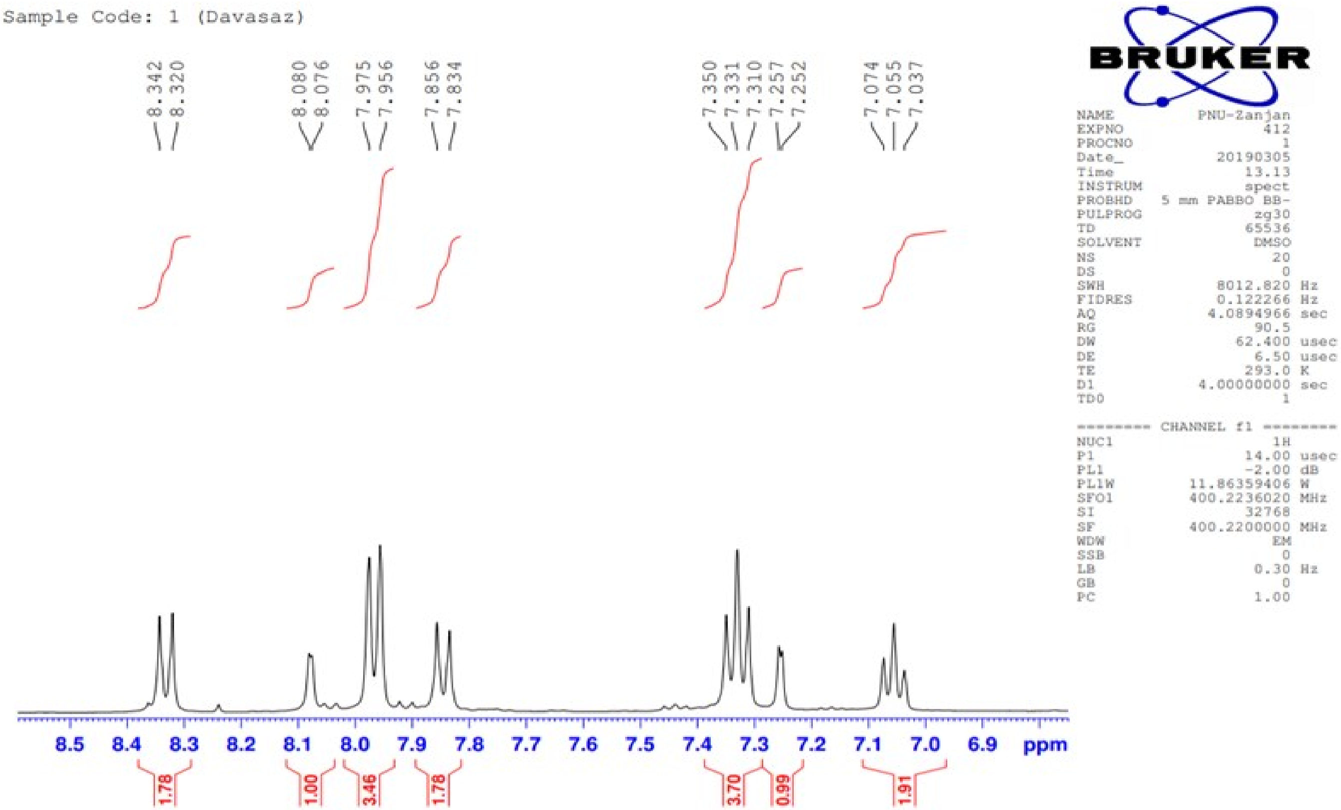

Fig ^{1}H NMR of **D2**

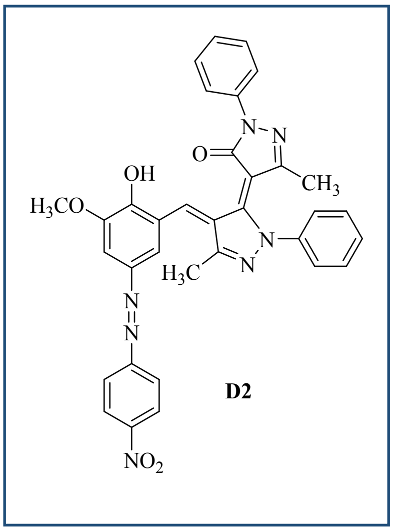

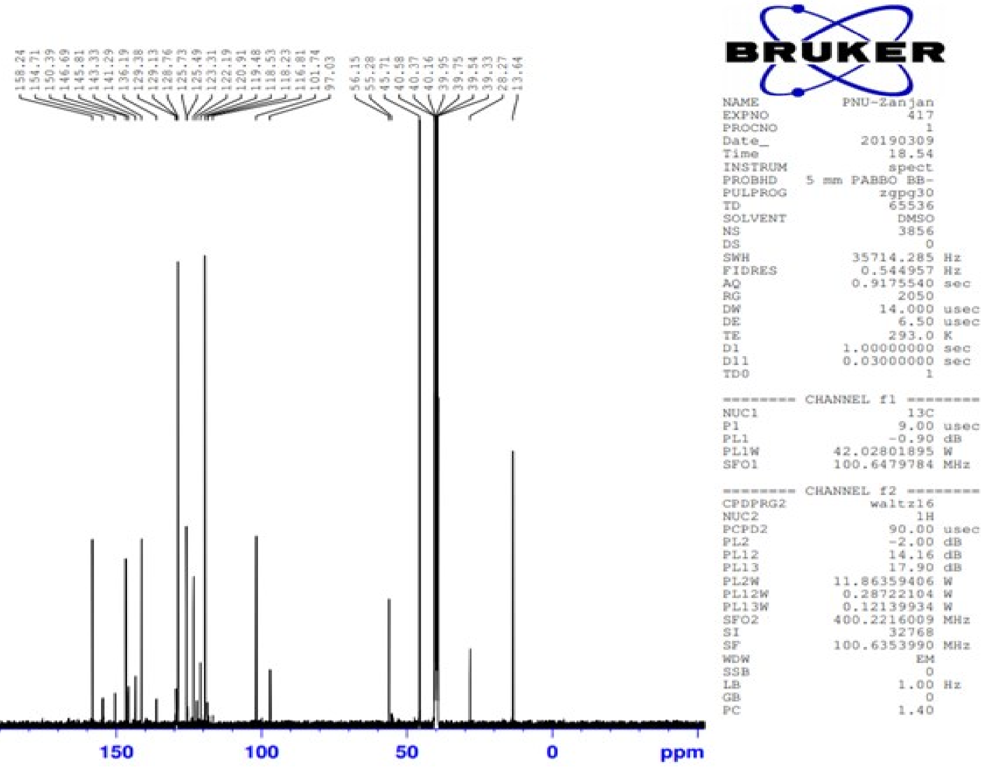

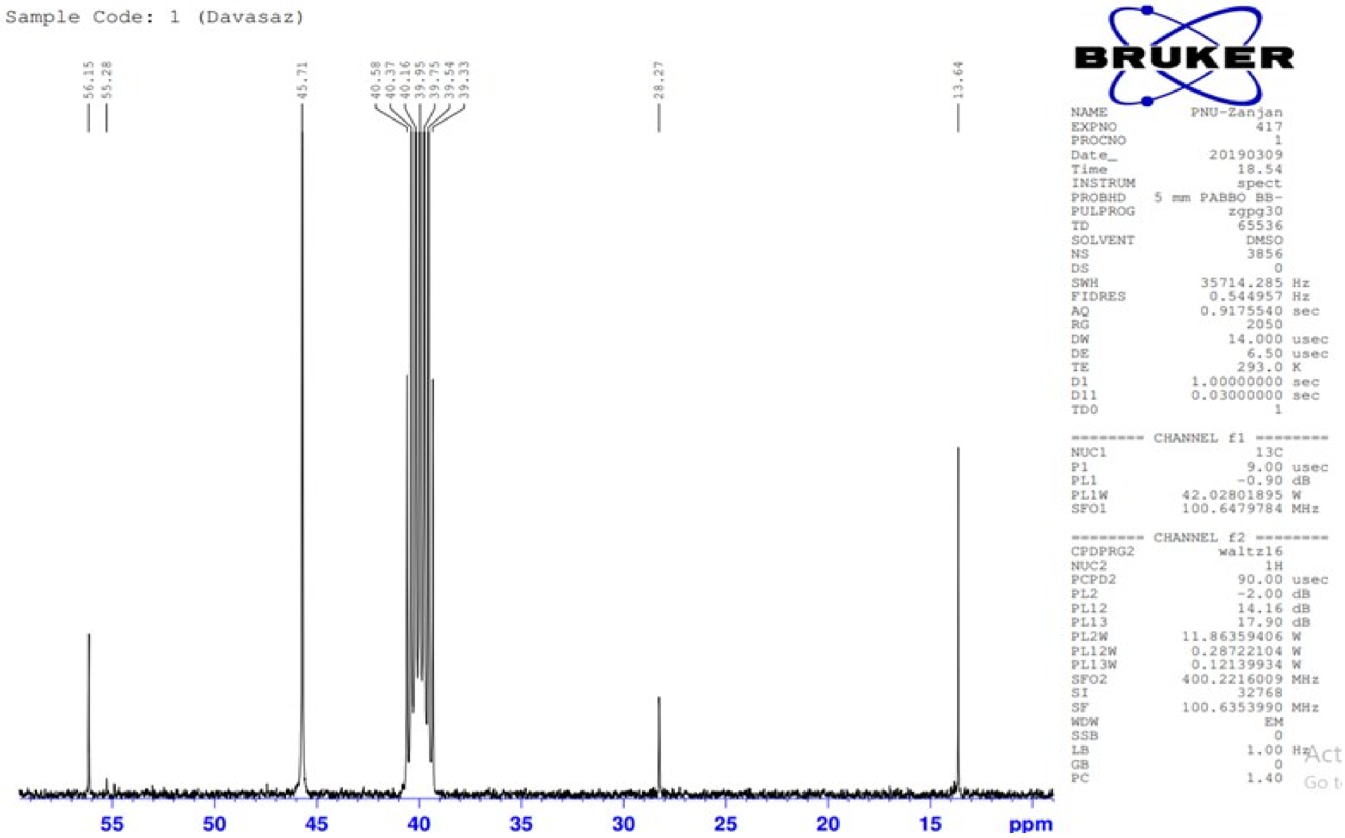

Fig ^{13}C NMR of **D2**.

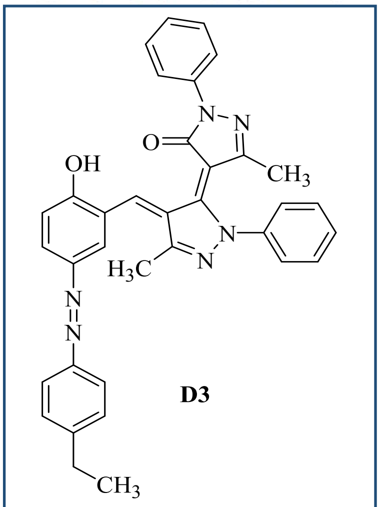

D3

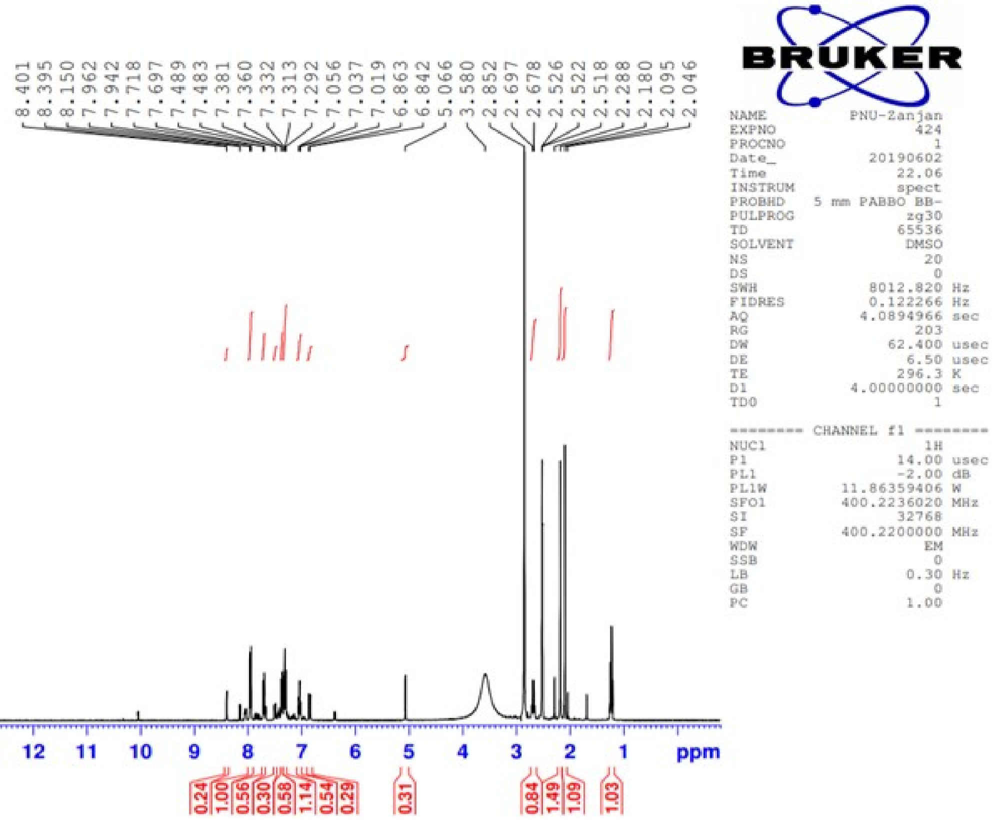

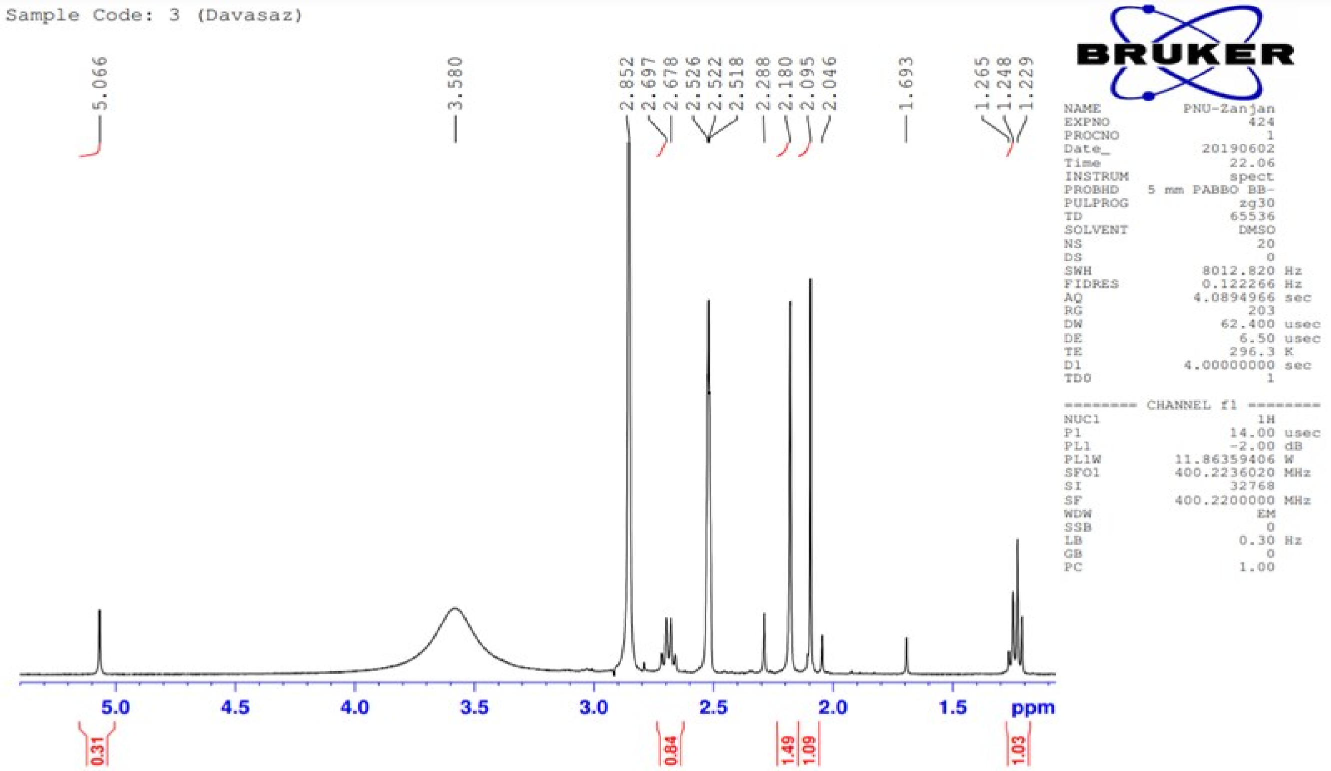

Fig ^{1}H NMR of **D3**.

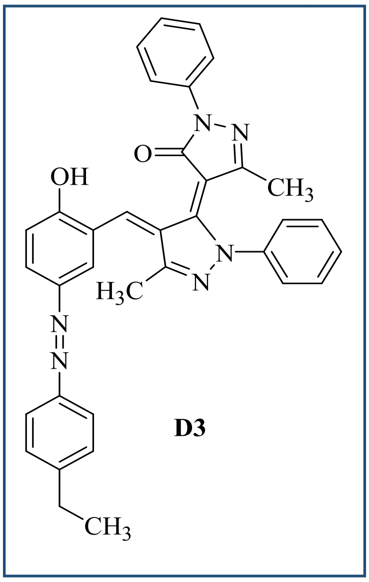

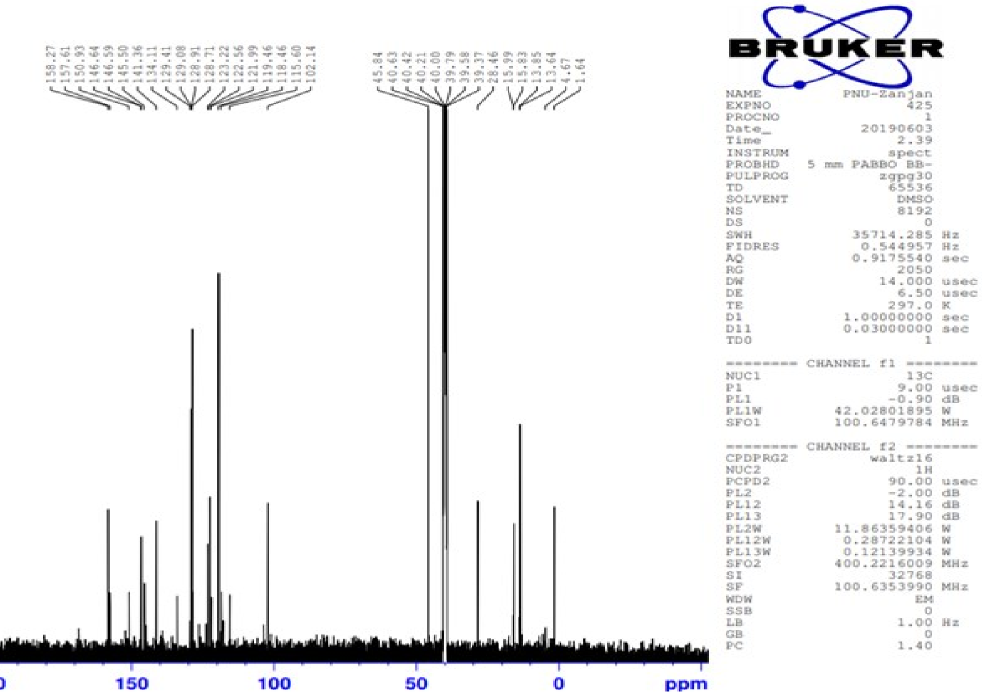

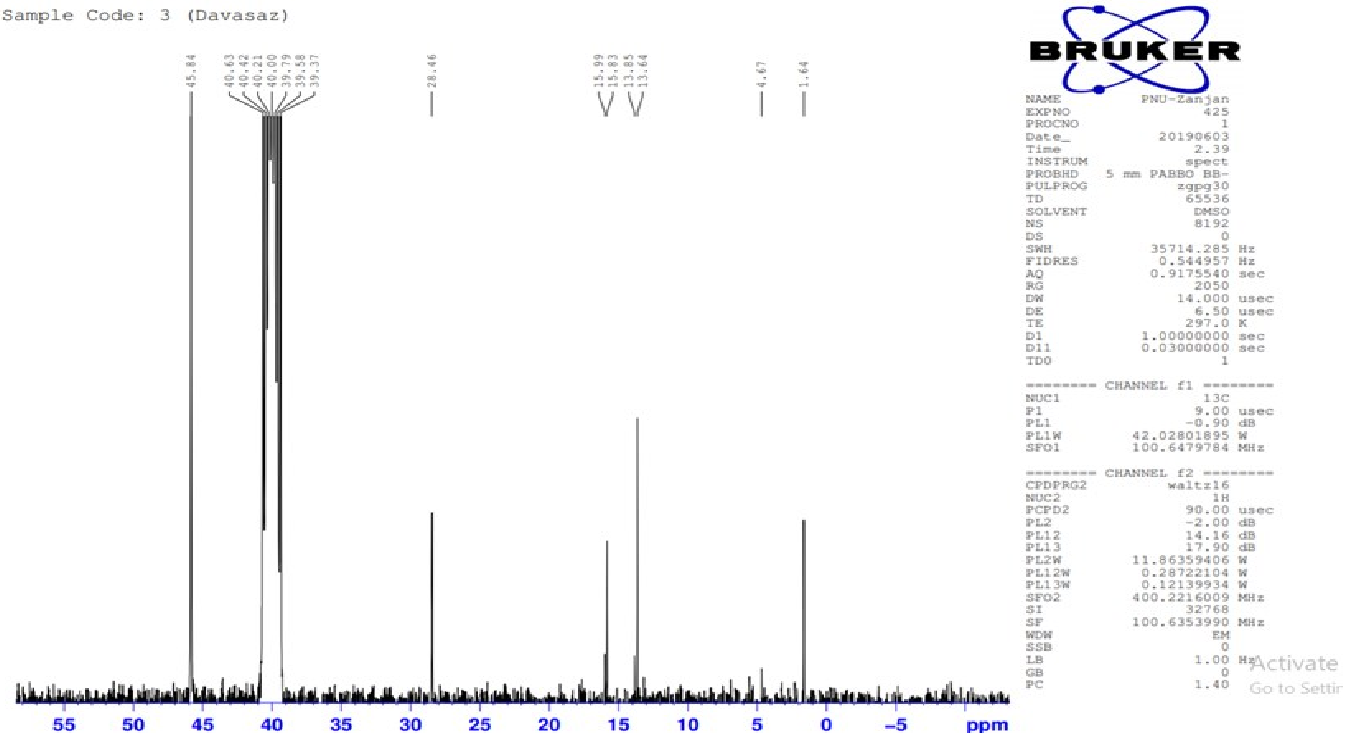

Fig ^{13}C NMR of **D3**.

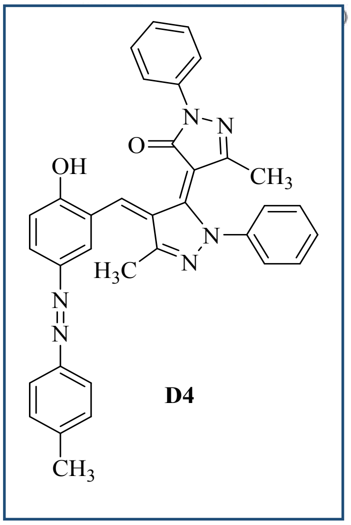

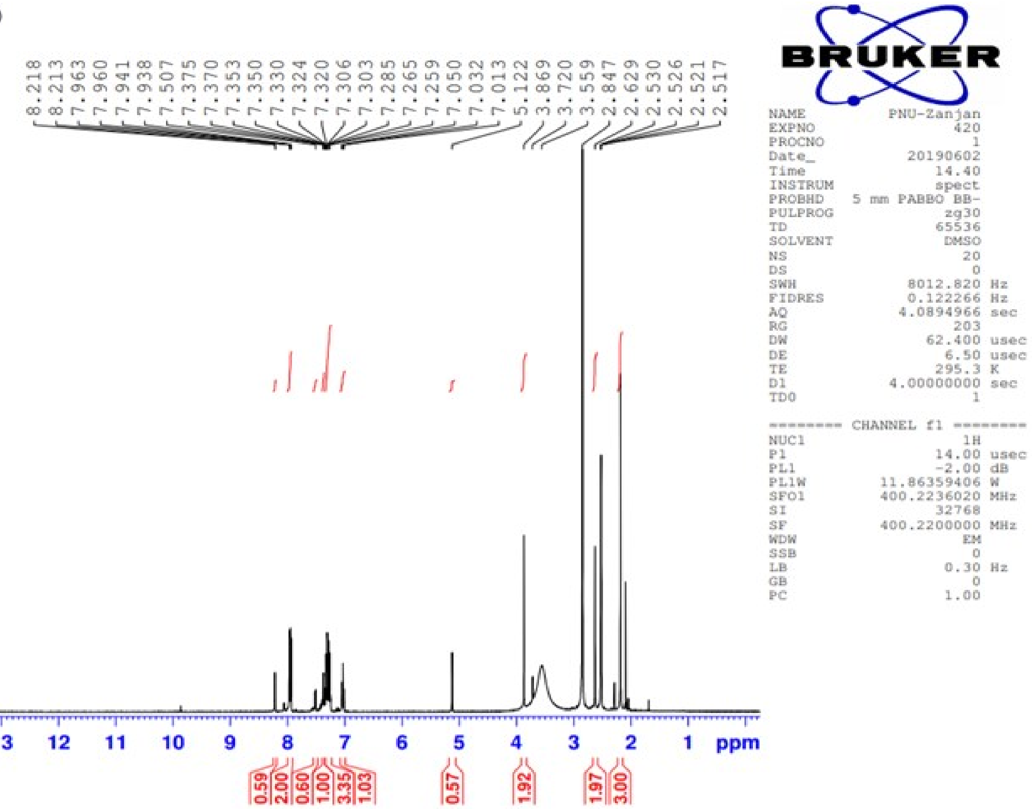

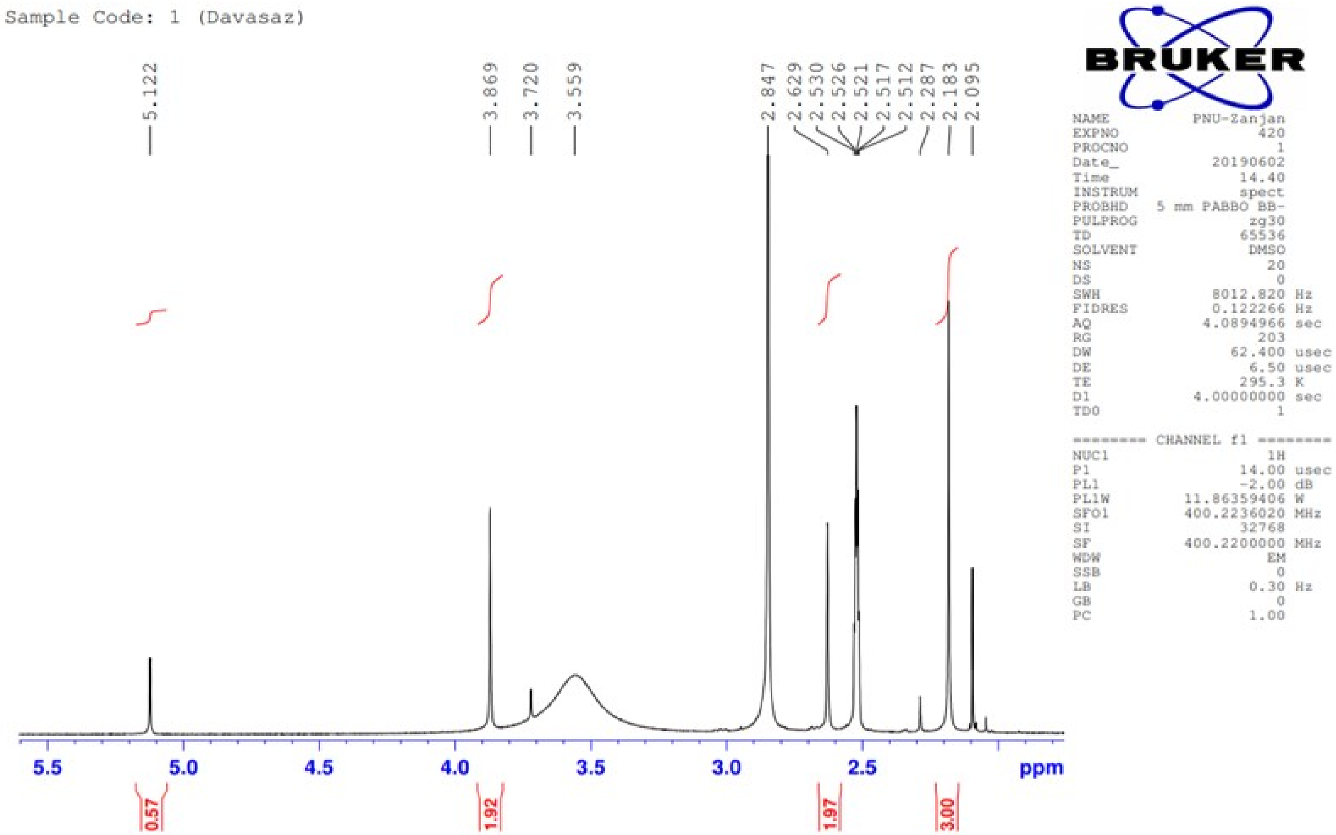

Fig ^{1}H NMR of **D4**.

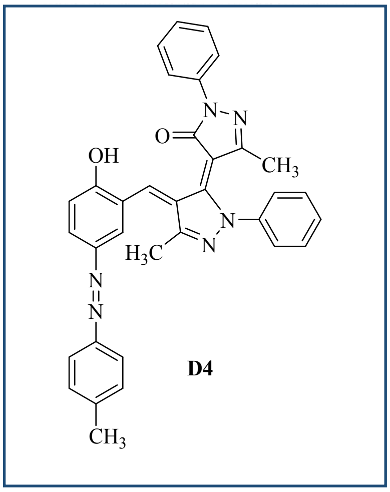

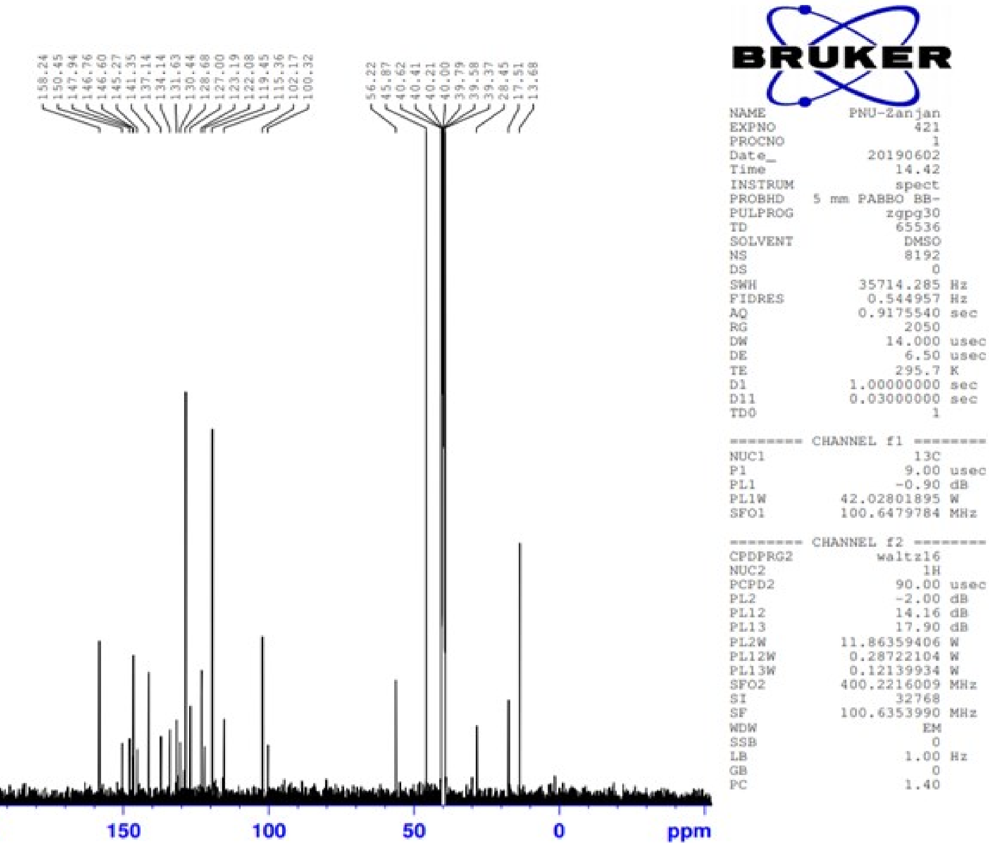

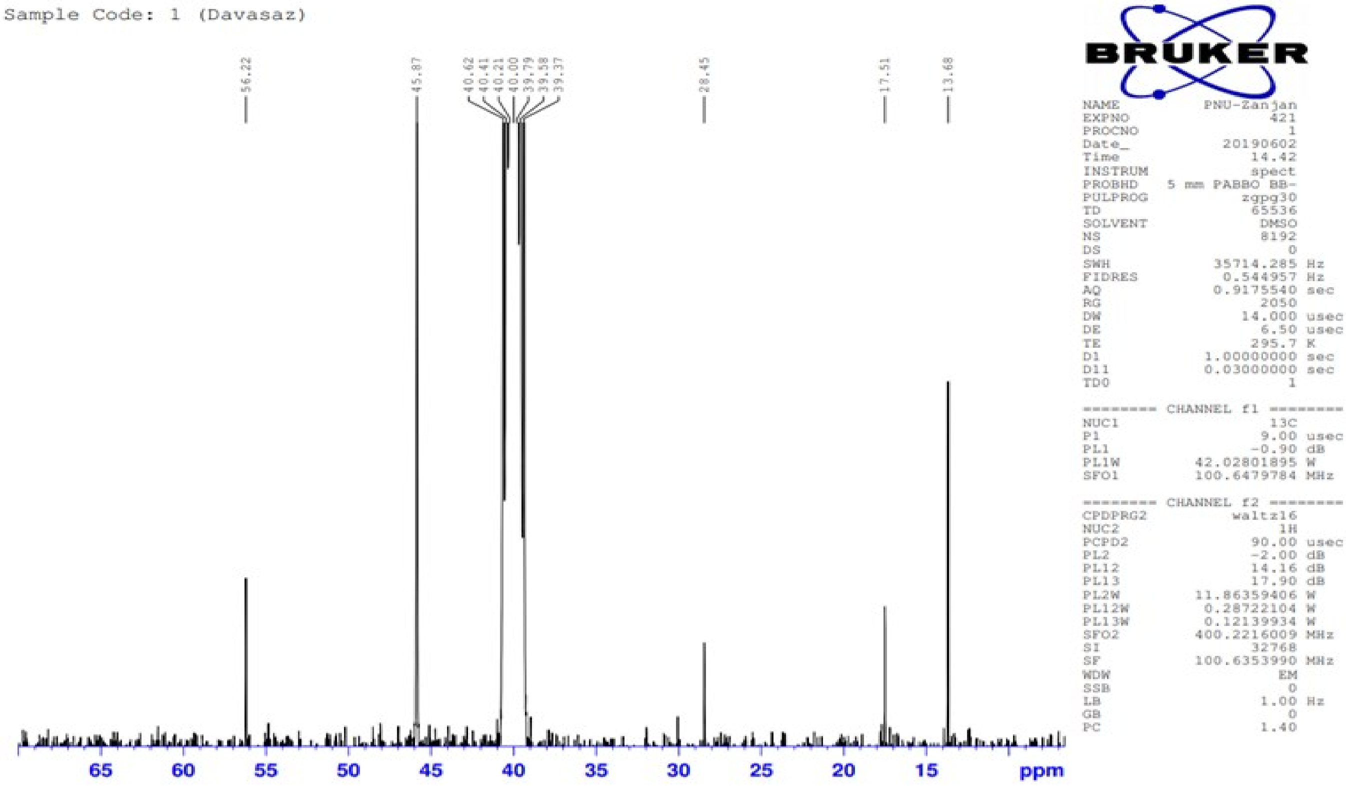

Fig ^{13}C NMR of **D4**.

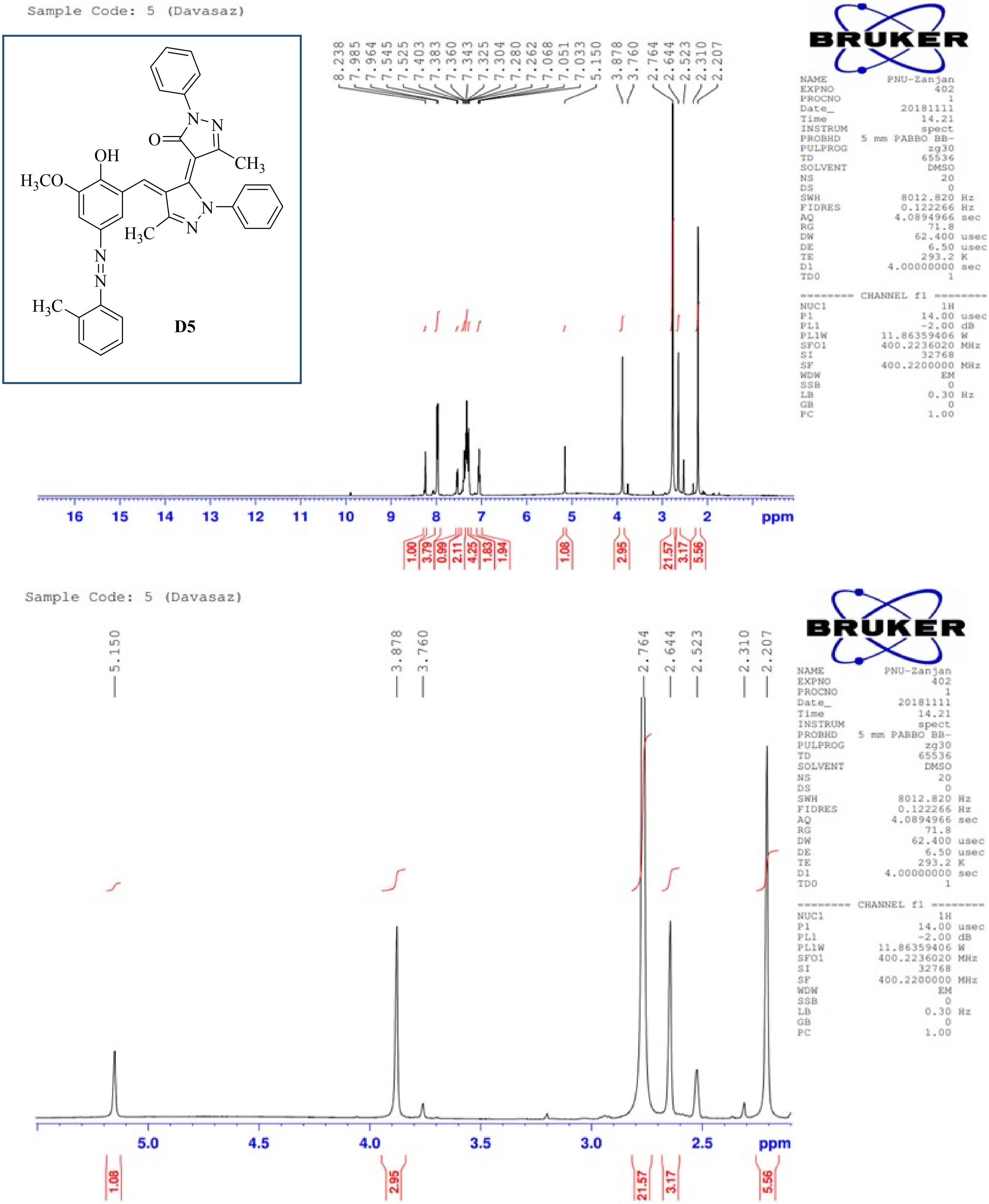

Fig ^{1}H NMR of **D5**

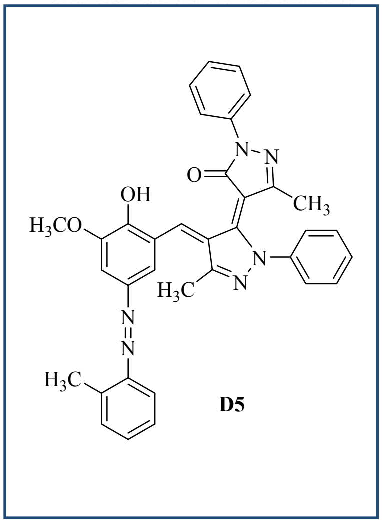

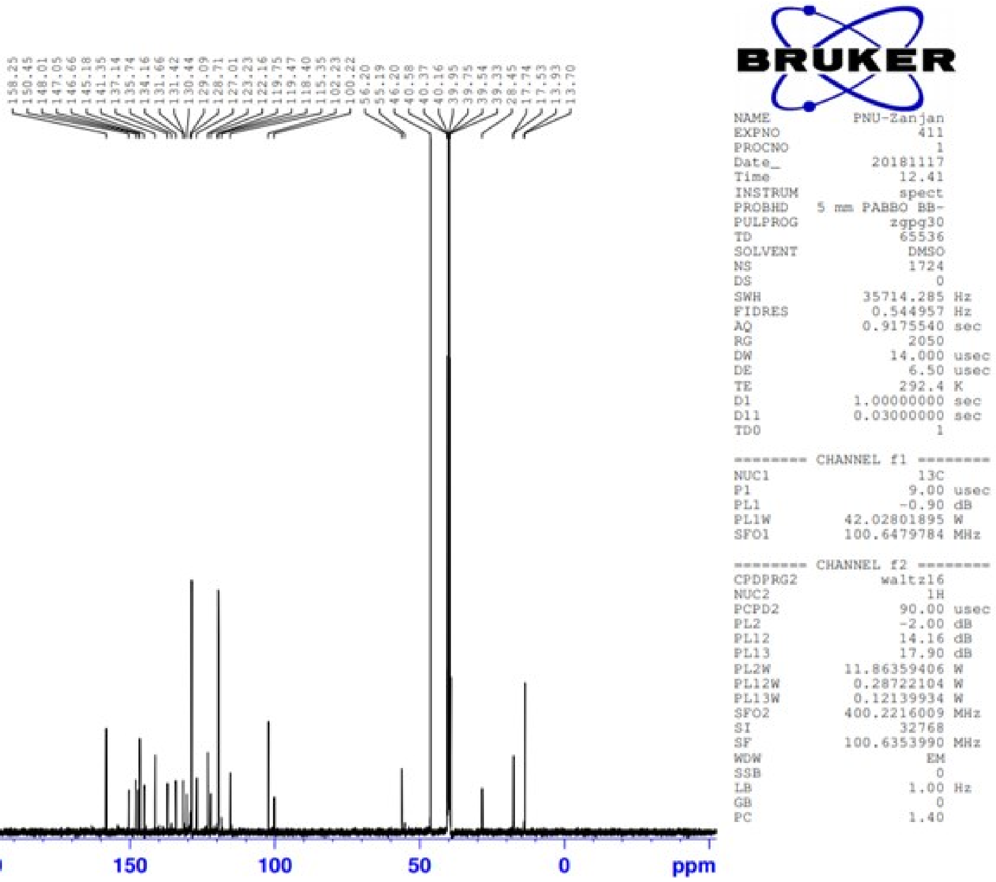

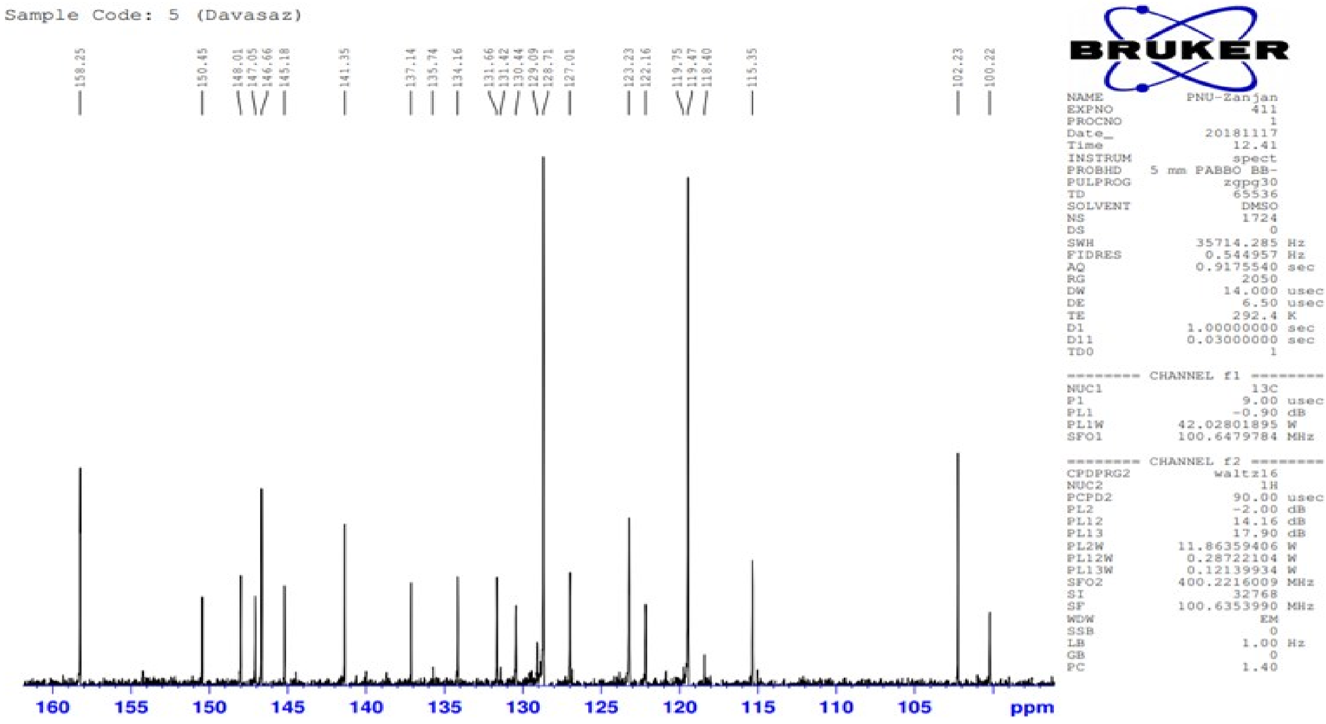

Fig ^{13}C NMR of **D5**.

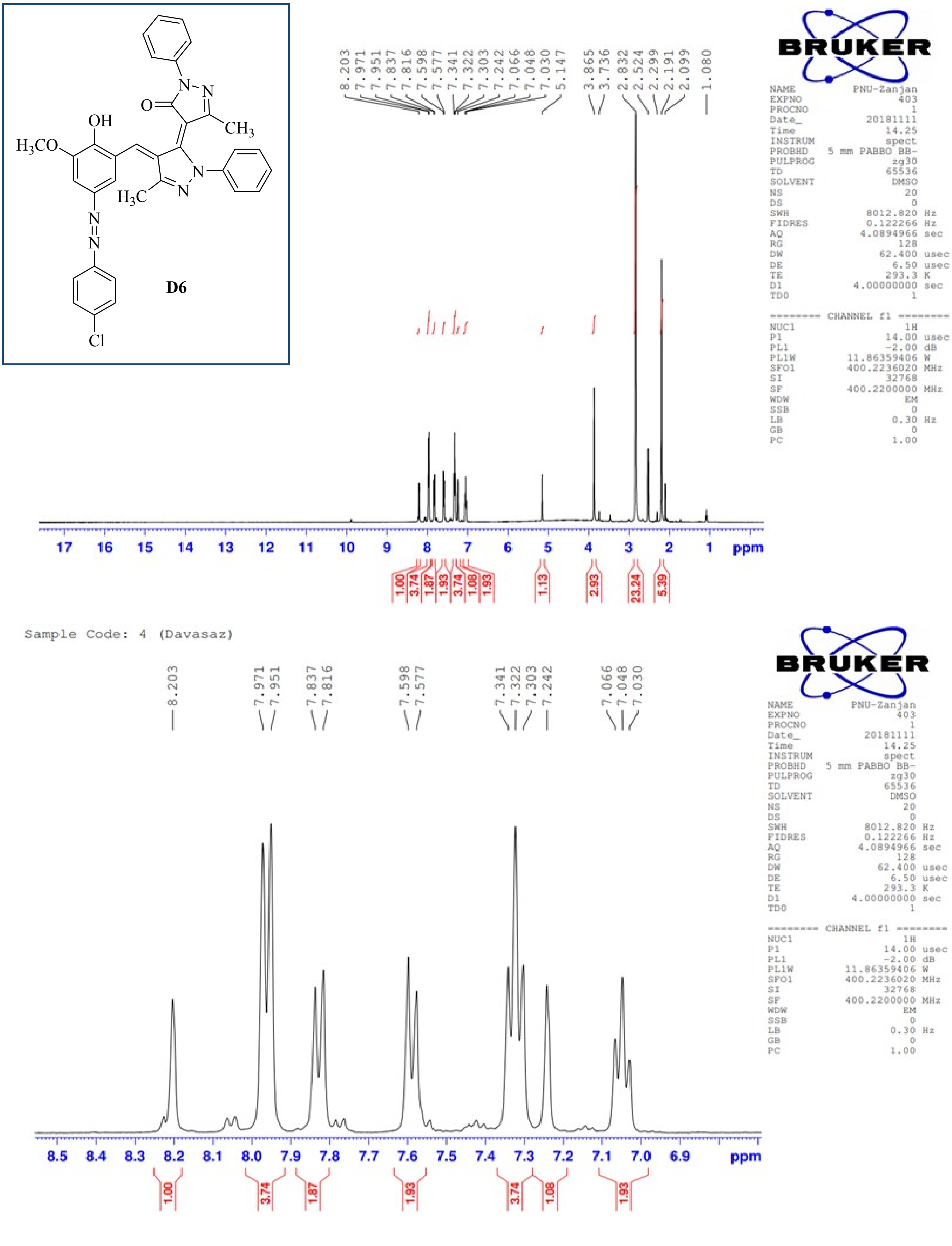

Fig ^{1}H NMR of **D6**

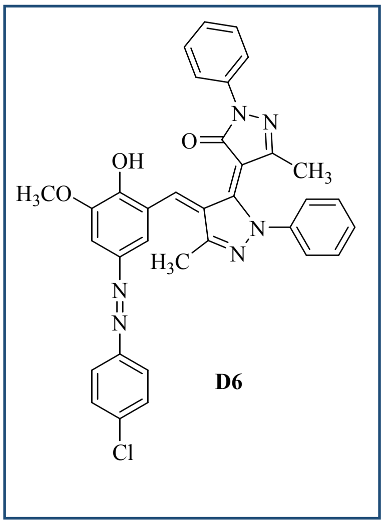

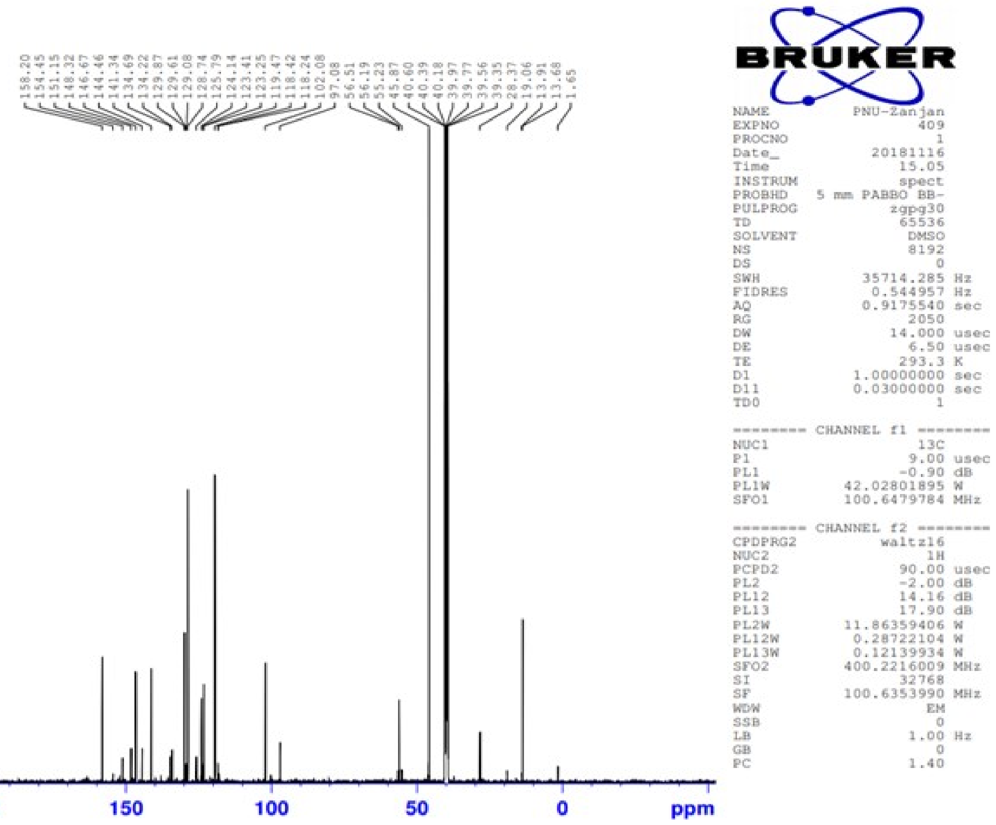

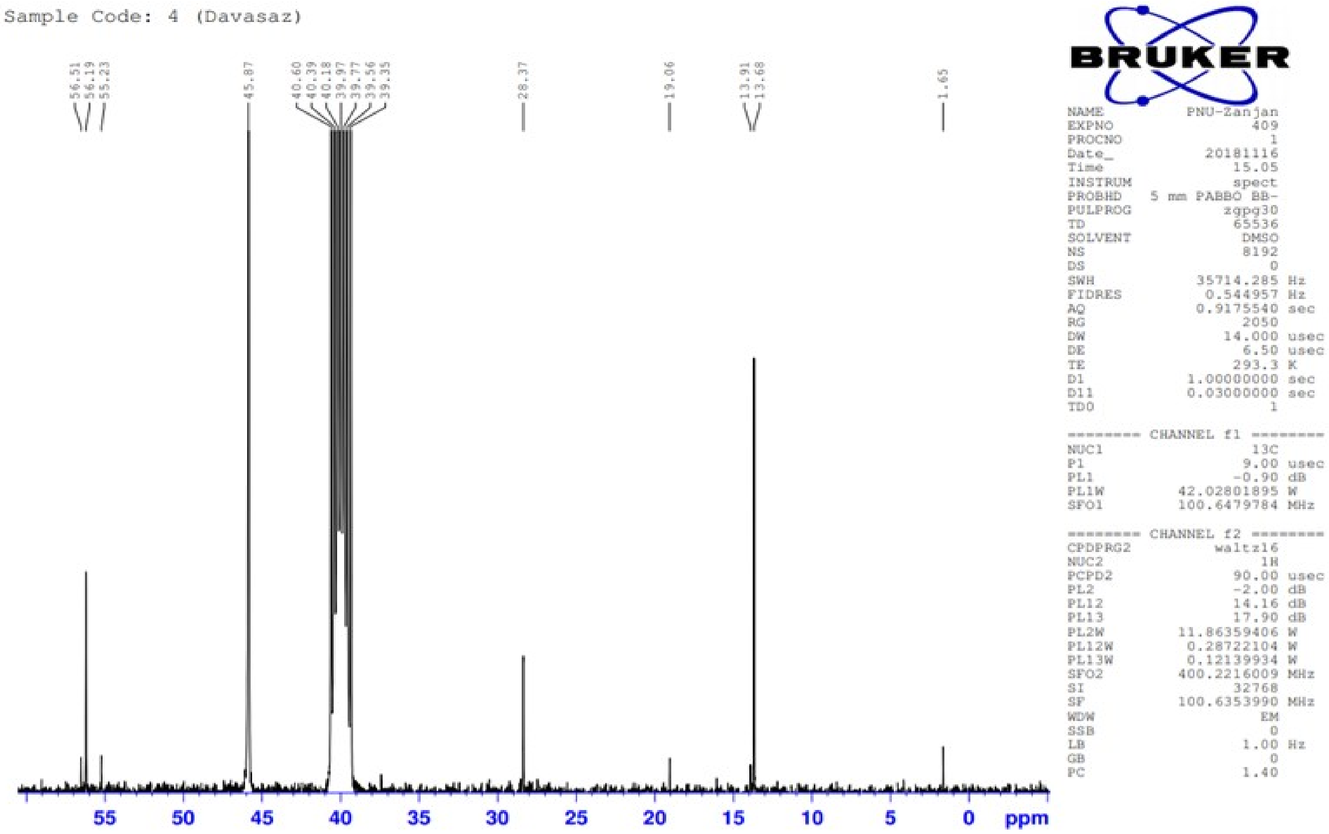

Fig ^{13}C NMR of D6.

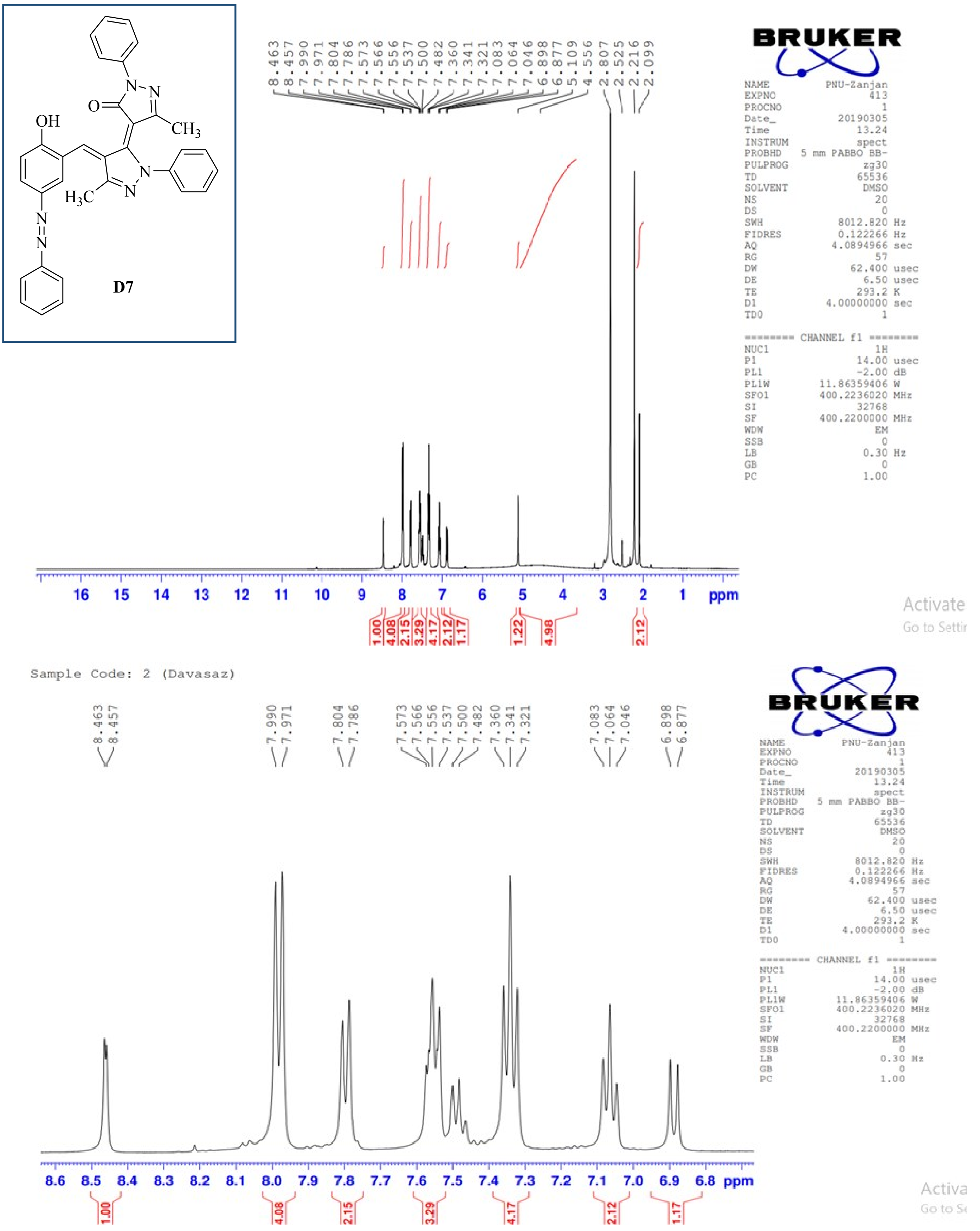

Fig ^{1}H NMR of D7.

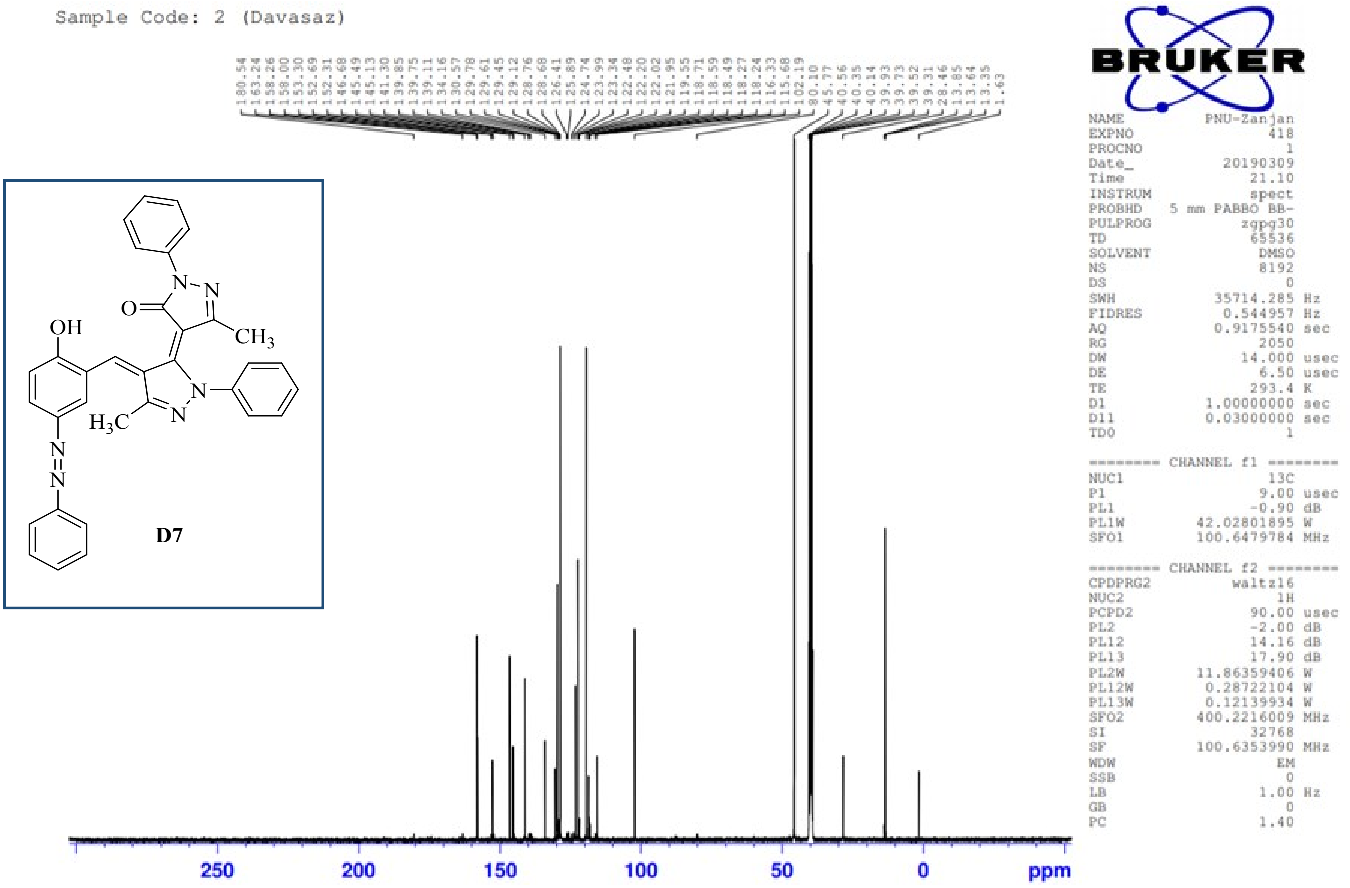

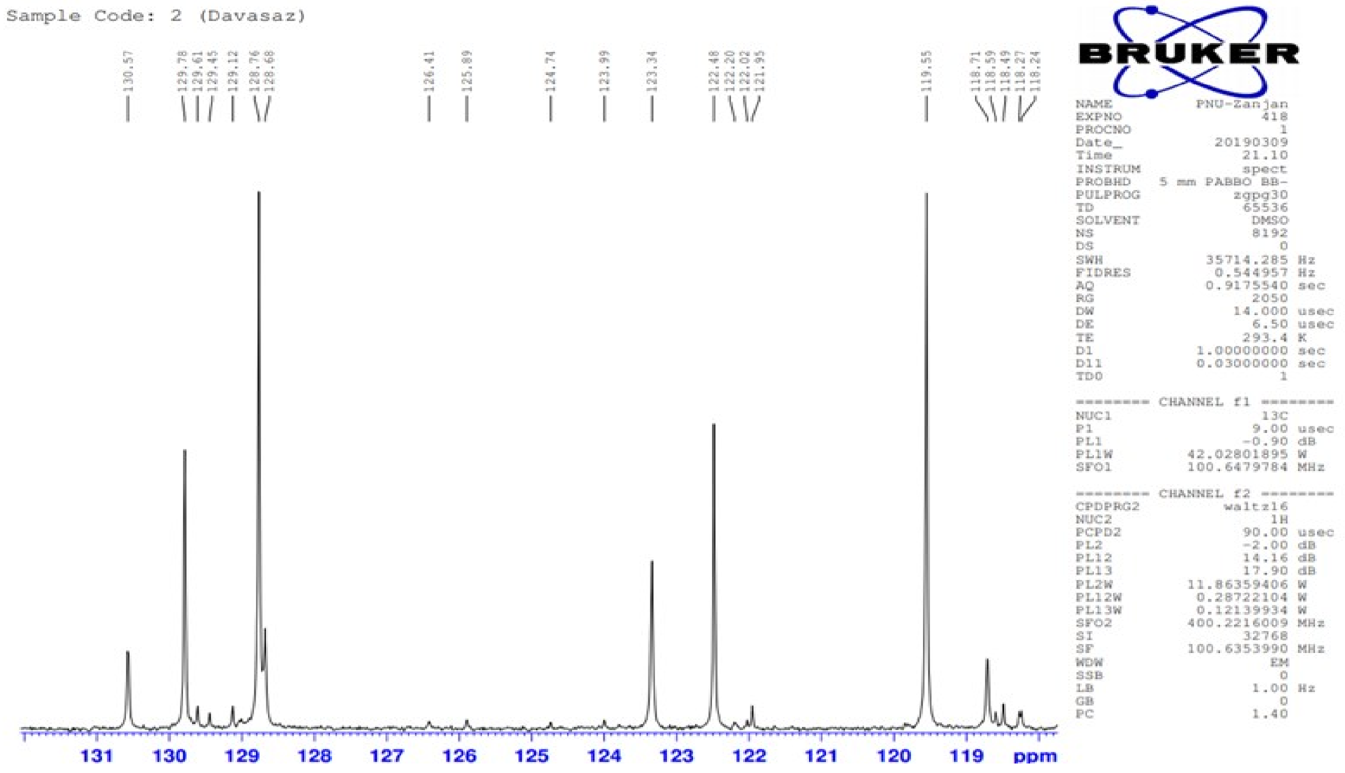

Fig ^{13}C NMR of D7.

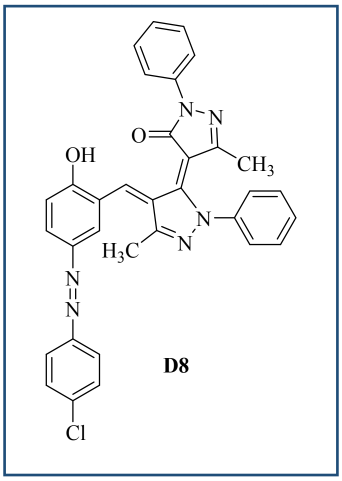

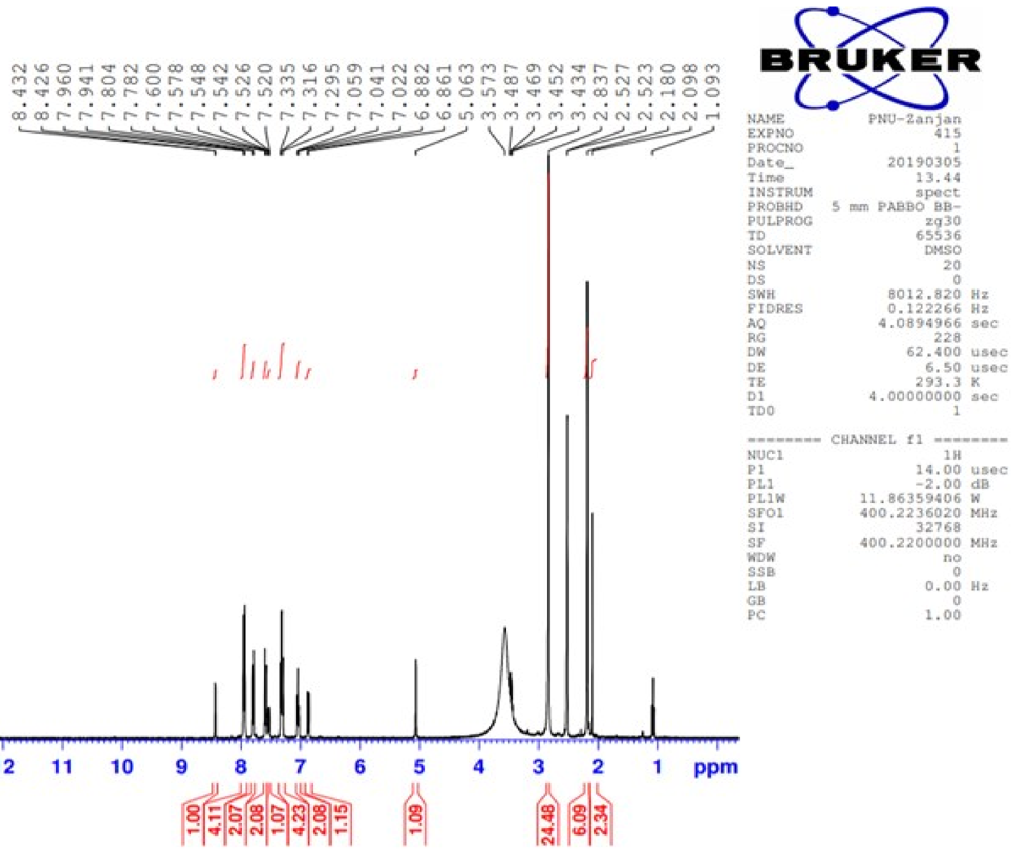

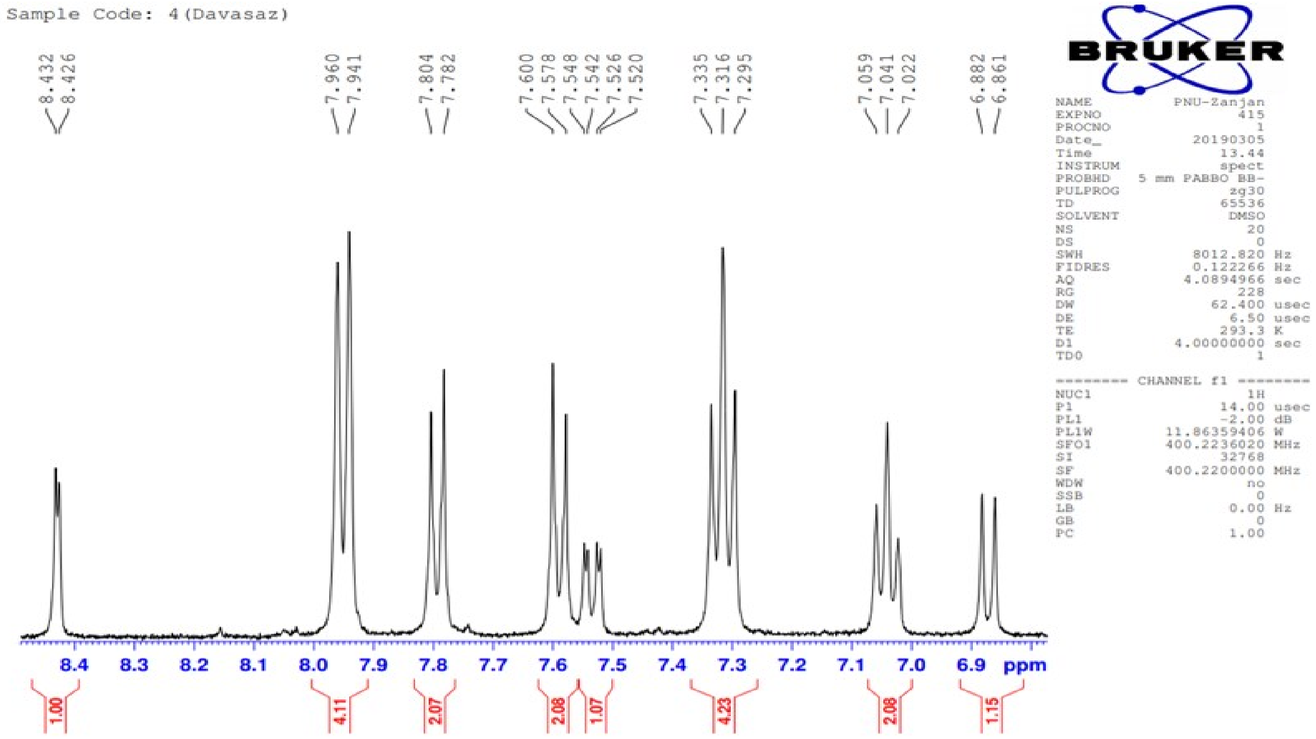

Fig ^{1}H NMR of **D8**.

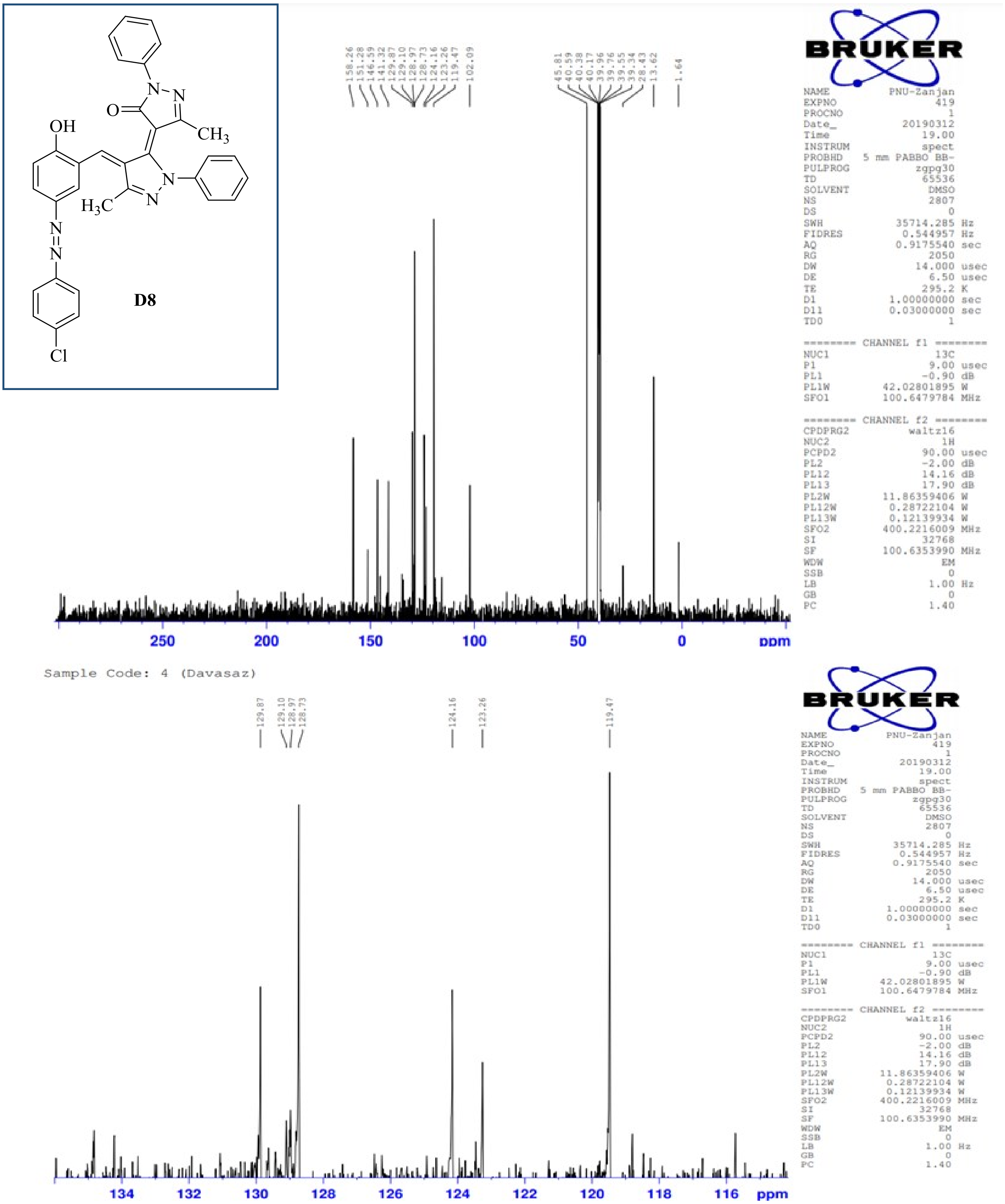

Fig ^{13}C NMR of **D8**.

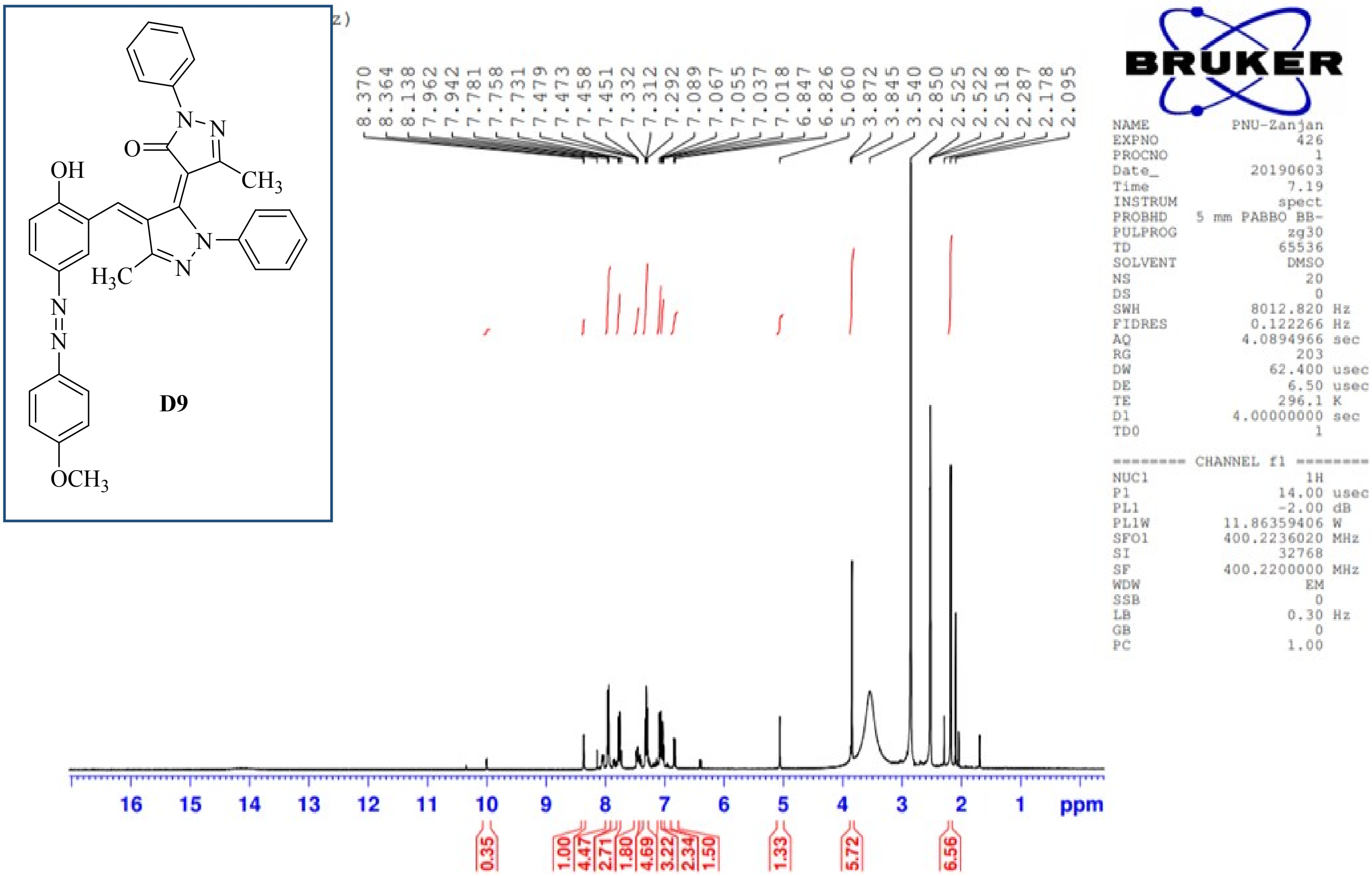

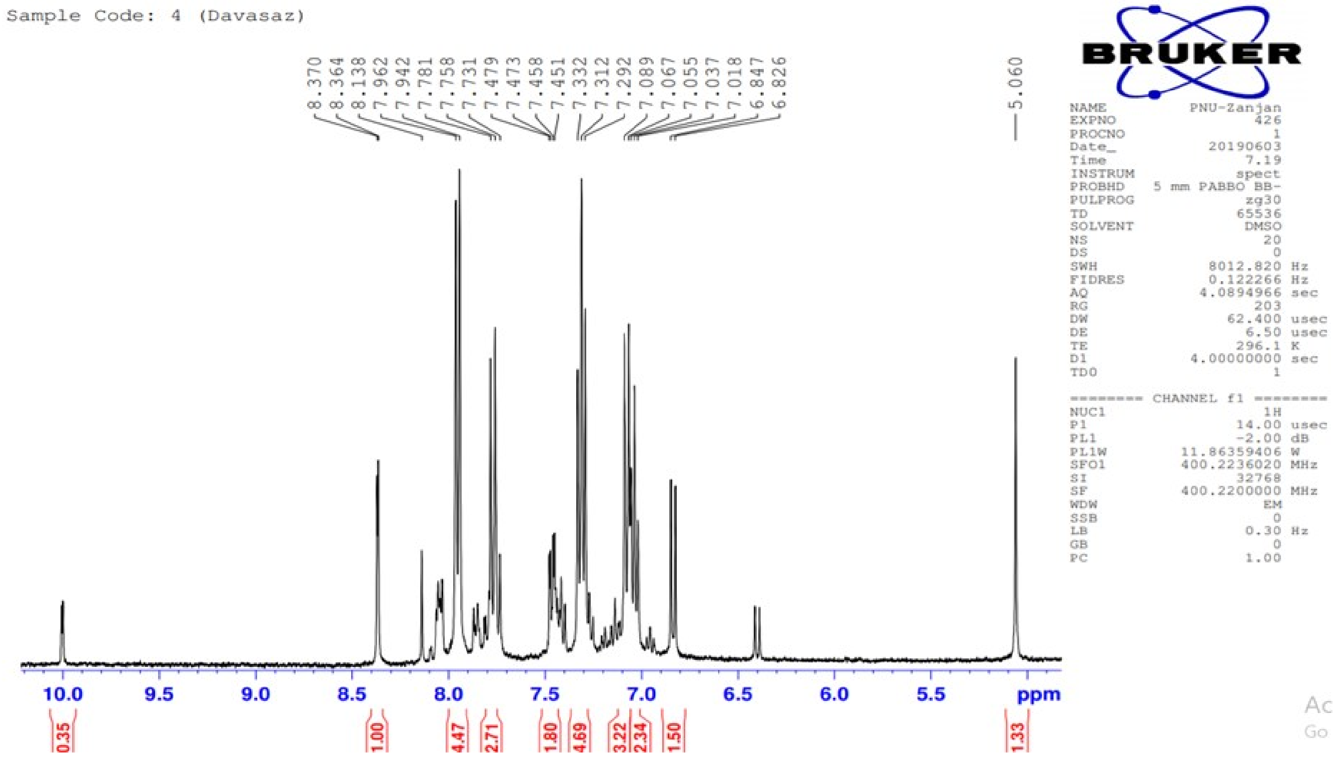

Fig ^{1}H NMR of **D9**.

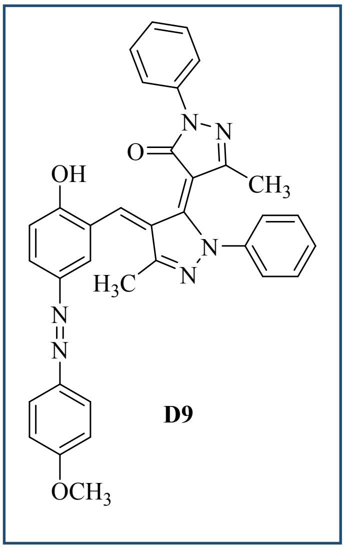

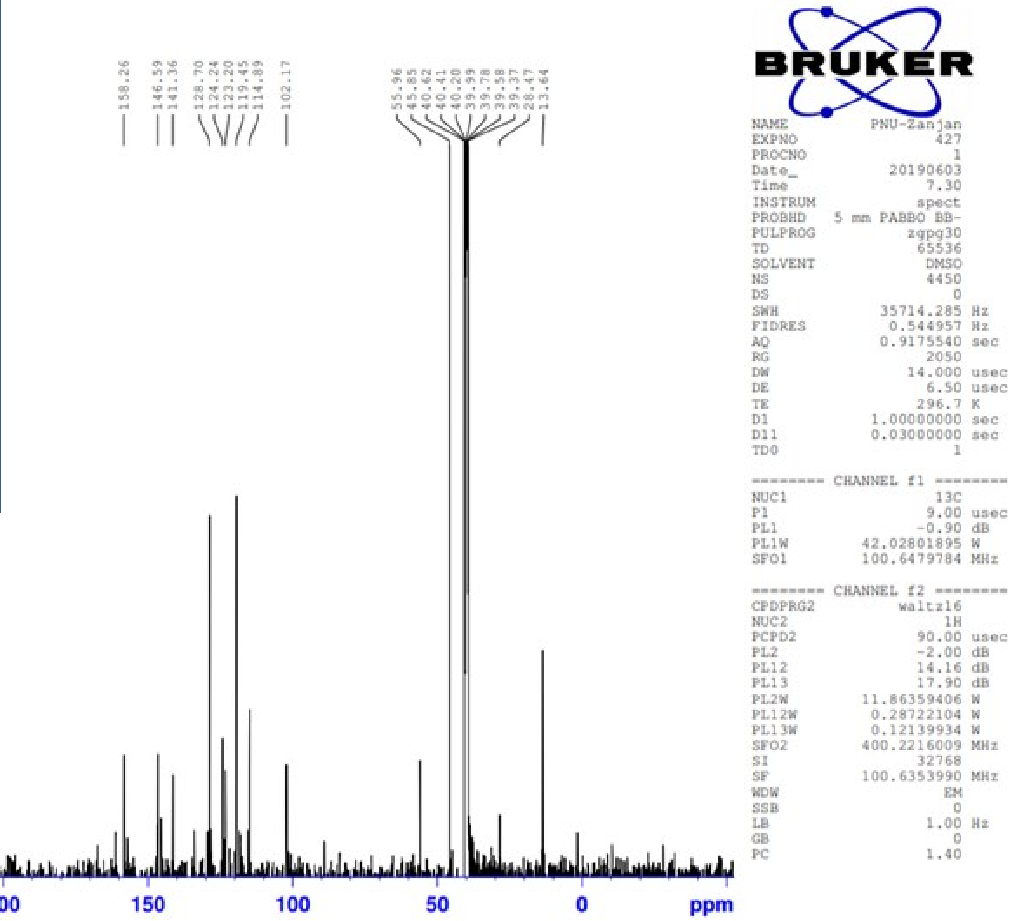

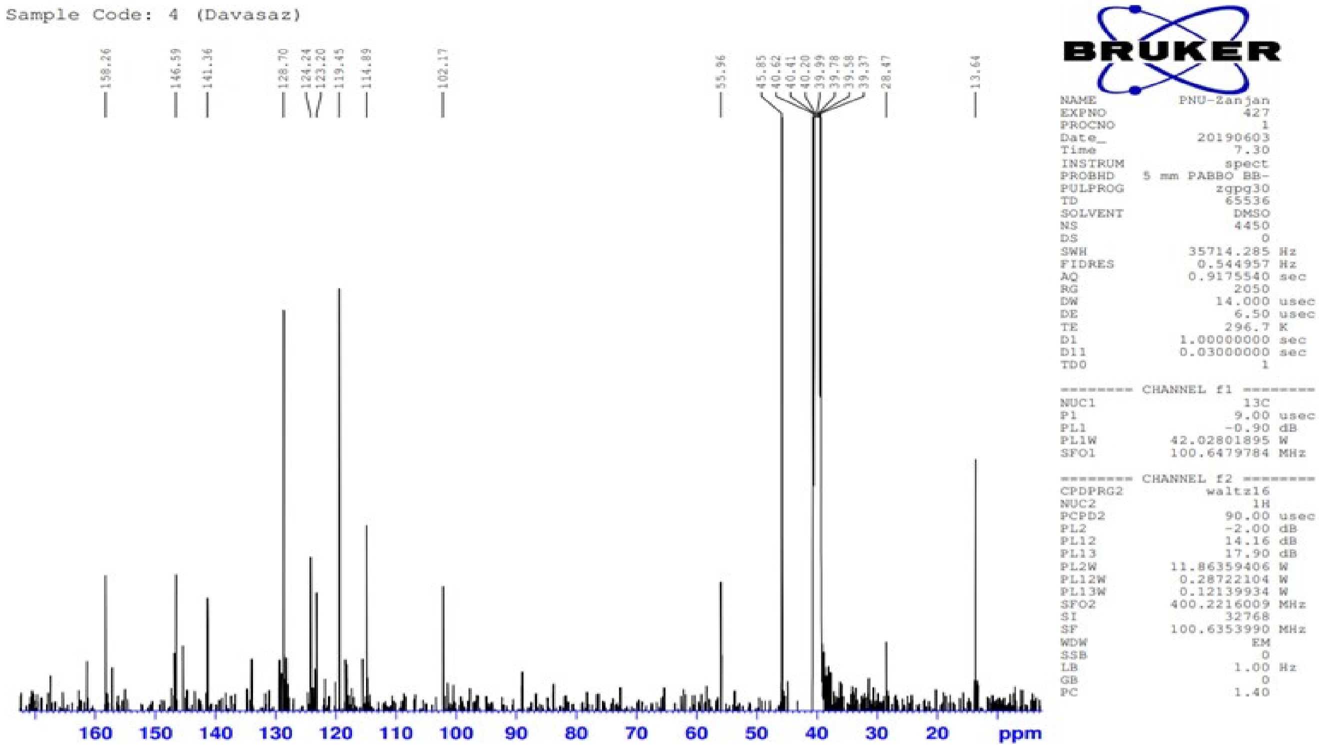

Fig ^{13}C NMR of **D9**.

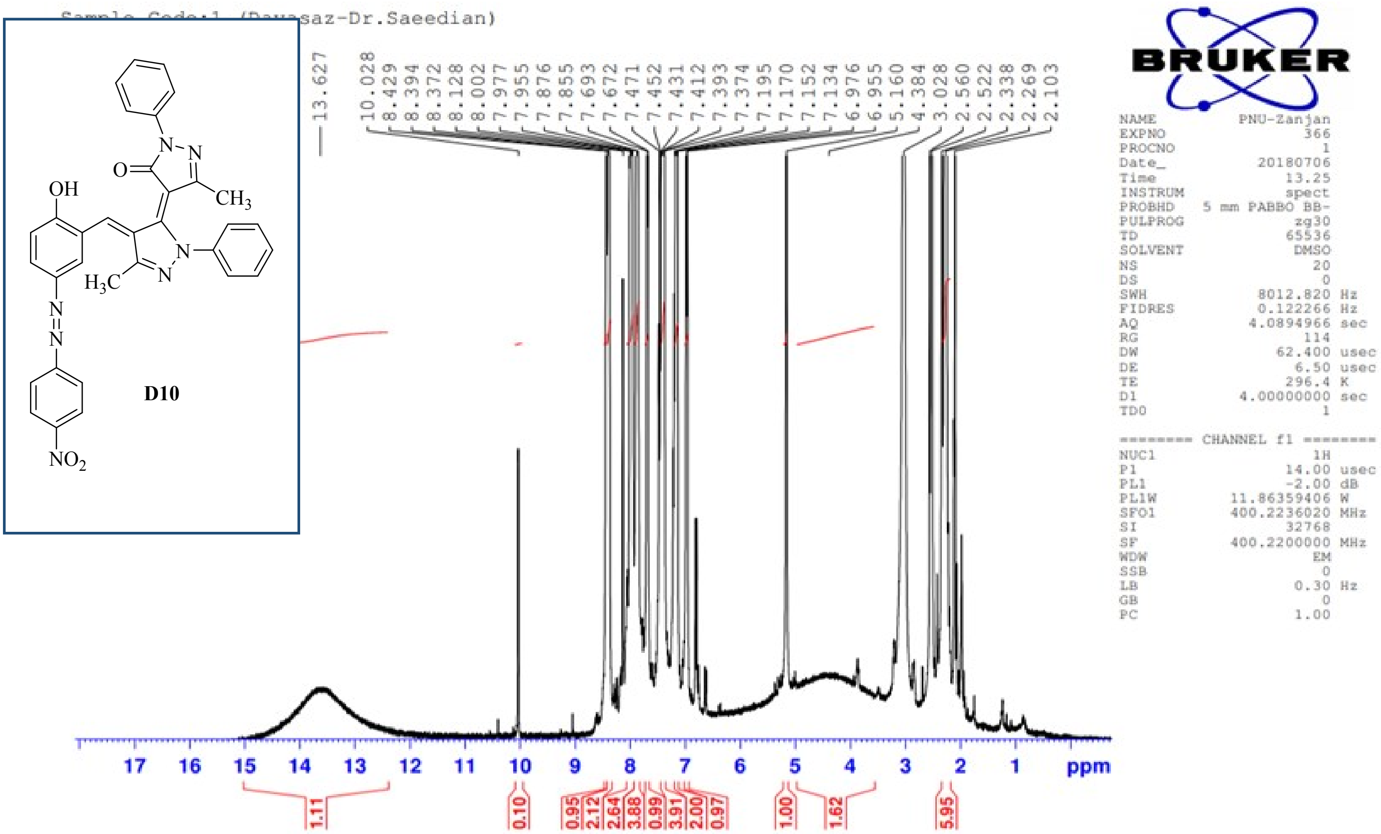

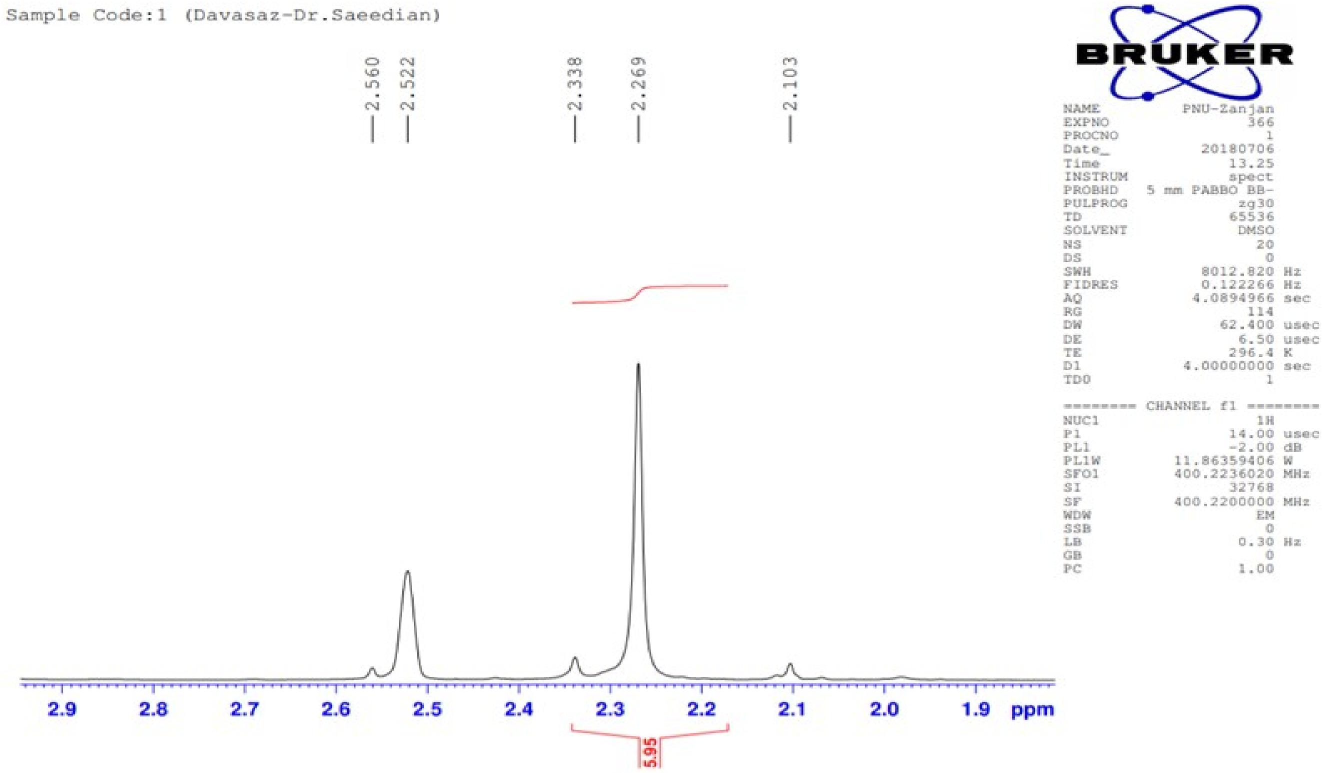

Fig ^{1}H NMR of **D10**

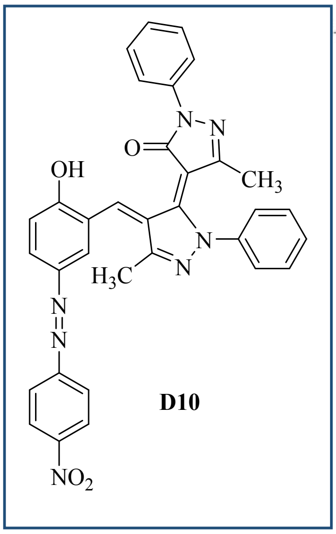

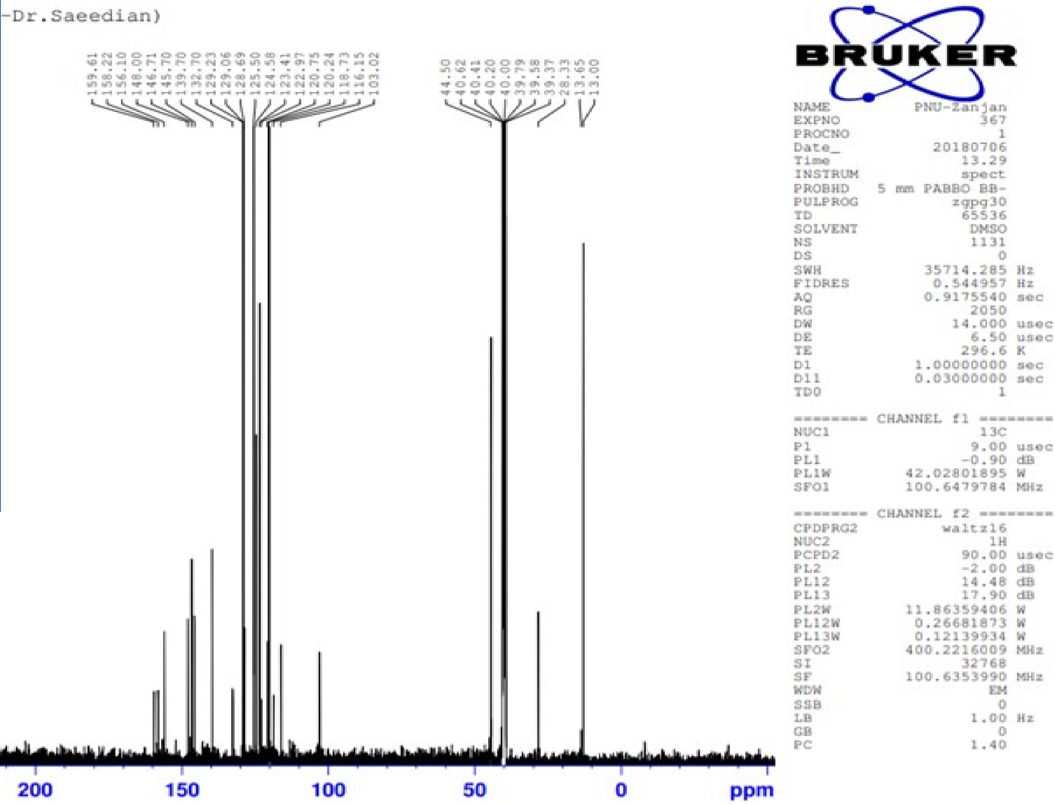

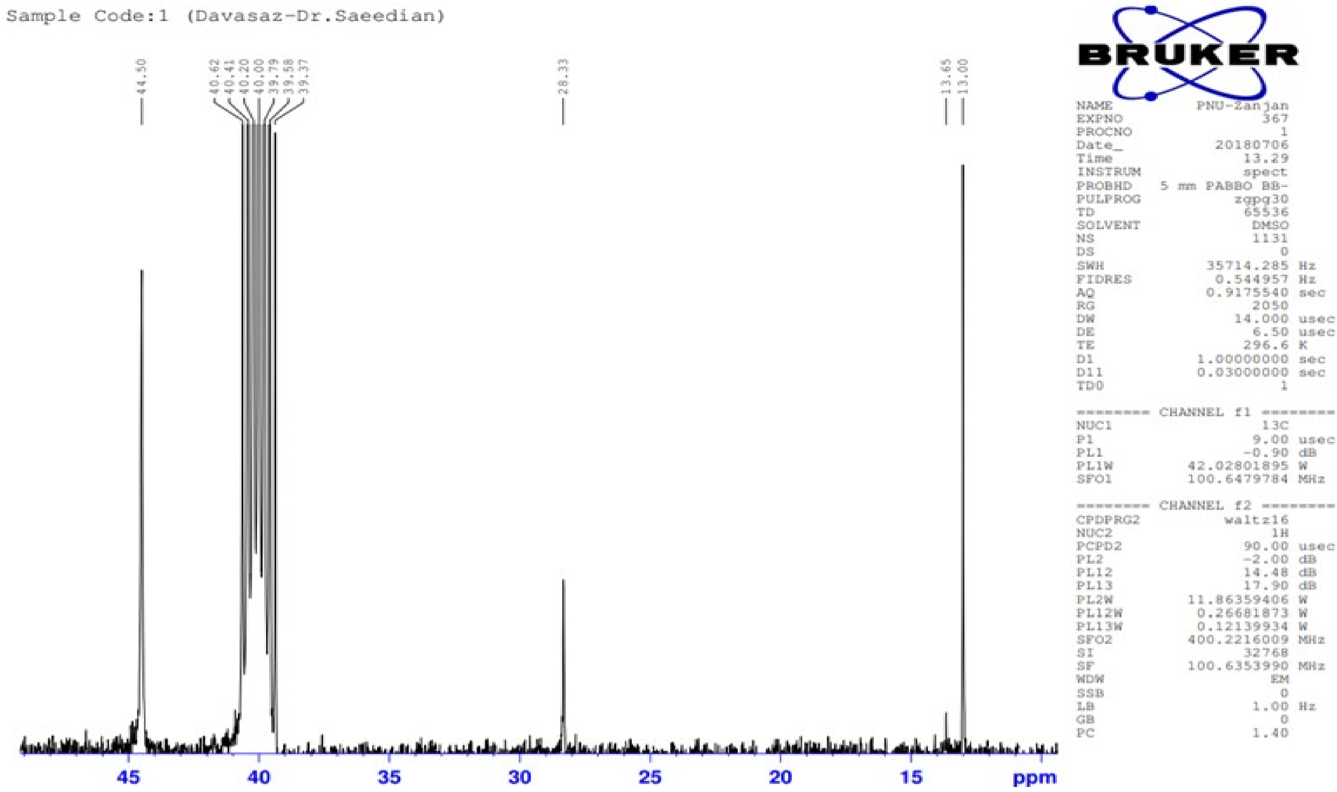

Fig ^{13}C NMR of D10.
